# The topology, stability, and instability of learning-induced brain network repertoires in schizophrenia

**DOI:** 10.1162/netn_a_00278

**Published:** 2023-01-01

**Authors:** Emmanuel D. Meram, Shahira Baajour, Asadur Chowdury, John Kopchick, Patricia Thomas, Usha Rajan, Dalal Khatib, Caroline Zajac-Benitez, Luay Haddad, Alireza Amirsadri, Jeffrey A. Stanley, Vaibhav A. Diwadkar

**Affiliations:** Department of Psychiatry and Behavioral Neurosciences, Brain Imaging Research Division, Wayne State University School of Medicine, Detroit, MI, USA

**Keywords:** Schizophrenia, Associative learning, fMRI, Graph theory, Betweenness centrality

## Abstract

There is a paucity of graph theoretic methods applied to task-based data in schizophrenia (SCZ). Tasks are useful for modulating brain network dynamics, and topology. Understanding how changes in task conditions impact inter-group differences in topology can elucidate unstable network characteristics in SCZ. Here, in a group of patients and healthy controls (*n* = 59 total, 32 SCZ), we used an associative learning task with four distinct conditions (Memory Formation, Post-Encoding Consolidation, Memory Retrieval, and Post-Retrieval Consolidation) to induce network dynamics. From the acquired fMRI time series data, betweenness centrality (BC), a metric of a node’s integrative value was used to summarize network topology in each condition. Patients showed (a) differences in BC across multiple nodes and conditions; (b) decreased BC in more integrative nodes, but increased BC in less integrative nodes; (c) discordant node ranks in each of the conditions; and (d) complex patterns of stability and instability of node ranks across conditions. These analyses reveal that task conditions induce highly variegated patterns of network dys-organization in SCZ. We suggest that the dys-connection syndrome that is schizophrenia, is a contextually evoked process, and that the tools of network neuroscience should be oriented toward elucidating the limits of this dys-connection.

## Author Summary

This paper demonstrates disordered network repertoires in schizophrenia during learning and memory. Graph theoretic analysis was applied to fMRI data collected during four distinct phases of a learning paradigm, to capture network repertoires. We show that the network topology changes across conditions, and that brain nodes peripheral to learning assumed greater importance in schizophrenia. Moreover, nodes with highly stable contributions to network topology were aberrantly localized to early sensory cortex in schizophrenia, but distributed across the brain in healthy participants. These results highlight the dynamic nature of the dys-connection syndrome, emphasizing the value of studying task-induced network topology in schizophrenia in conjunction with graph theoretic metrics.

## INTRODUCTION

Schizophrenia is a complex and debilitating neuropsychiatric disorder that has long been characterized as a “dys-connection syndrome” of the brain (Friston, Brown, Siemerkus, & Stephan, 2016; Silverstein, Bressler, & Diwadkar, 2016). Disordered brain network repertoires are characteristic signatures of dys-connection (Spronk et al., 2021), indicative of a profound loss of functional integrity that the disorder induces. The current investigation was motivated by two interrelated goals: (a) to characterize altered brain network repertoires in schizophrenia evoked during each of four distinct conditions of an associative learning paradigm (Baajour et al., 2020; Stanley et al., 2017), by (b) estimating the betweenness centrality (BC) (Rubinov & Sporns, 2010; Telesford, Joyce, Hayasaka, Burdette, & Laurienti, 2011) of each cerebral node. BC, a graph theoretic measure, is well suited to capture the integrative profile of a node in any directed or undirected graph. In our case, nodes represent brain regions and vertices represent the condition-evoked “distance” (i.e., estimated functional connectivity) between those regions.

In schizophrenia, graph theoretic measures have been widely used to capture altered network repertoires derived from resting-state fMRI signals (J. Chen et al., 2021; Ji et al., 2019; Lei et al., 2020; Rolls et al., 2020). More recent applications have used graph theory to investigate altered task-driven connectomics. These investigations have been conducted in domains including working memory (Deng et al., 2022; Stolz, Emerson, Nahkuri, Porter, & Harrington, 2021; Yang et al., 2020), executive function (Zhao, Cheng, Li, & Yu, 2018), social cognition (Oliver et al., 2021), and theory of mind (Bitsch, Berger, Nagels, Falkenberg, & Straube, 2021). Task-driven fMRI is valuable in explicitly driving network dynamics, thus promoting discovery of contextually driven network differences (Baajour et al., 2020; Thakkar, Diwadkar, & Rolfs, 2017; Tso et al., 2021). Contextual modulation may be particularly valuable in understanding schizophrenia, because functional dys-connection is like to be contextually evoked, rather than invariant across behavioral contexts (Logothetis, 2008; Stephan & Roebroeck, 2012). Our own reliance on an object-location associative learning paradigm (Büchel, Coull, & Friston, 1999; Diwadkar et al., 2008) was motivated by (a) the knowledge that impaired learning interjects with the schizophrenia phenotype (Brambilla et al., 2011; Brambilla, Riva, Melcangi, & Diwadkar, 2007; Stephan, Baldeweg, & Friston, 2006; Wannan et al., 2018), and (b) the ability to configure the learning paradigm into distinct conditions related to Memory Formation (Encoding), Post-Encoding Consolidation (requiring recapitulation of associations presented), cued Memory Retrieval (retrieval of object name at the cued location), and Post-Retrieval Consolidation. While the process of learning is distributed across conditions, each condition provides unique characteristics to the learning process. Thus, Memory Formation and Retrieval are performance driven, but Consolidation while devoid of sensorimotor stimulation is an active rest state, requiring memories to be rehearsed and recapitulated (Ravishankar et al., 2019). Accordingly, we expect network repertoires summarized by BC (Bullmore & Sporns, 2009) to reflect condition-driven processing. Inter-group differences are unlikely to be condition independent.

Graph theoretic approaches can efficiently summarize swathes of spatiotemporal fMRI data (Farahani, Karwowski, & Lighthall, 2019), and multiple metrics have been proposed for capturing the different roles played by a node in modulating the “flow” of information in directed or undirected graphs (van den Heuvel & Sporns, 2013). These metrics range from “basic” measures of degree centrality including in- and out-degree centrality (that respectively capture the number of vertices entering or exiting a node), to more “integrative” measures such as closeness centrality (CC) (Bavelas, 1950) and BC. Integrative metrics are designed to capture a node’s role based on its cumulative relationships with other nodes in a network. CC is calculated as the reciprocal of the sum of the length of the shortest paths between the node and all other nodes in the graph, and while similar to BC, has typically been employed with binary as opposed to weighted graphs. BC, however, is more sensitive to the integrative value of a node precisely because it quantifies how a node acts as a bridge along the shortest path between any two other nodes, a measure that is a central element of a node’s functional role. Thus, BC provides an excellent representation of a node’s relative importance within a network and has been used as an index of a node’s “hubness” (Rubinov & Sporns, 2010) or integrative value (Kivimaki, Lebichot, Saramaki, & Saerens, 2016). Understanding how a highly integrative task like associative learning impacts the integrative roles of nodes was central to our motivations, providing the rationale for our choice of BC in characterizing network repertoires.

Across participants and conditions, the edges between nodes in each graph were represented by a typical distance metric (using bivariate correlation models applied to summarize time series data) (Wang et al., 2015) (resulting in a weighted, undirected graph). Then, BC was estimated for each node in each participant and condition. Subsequent analyses were directed toward understanding (a) how each of the four task conditions modulated the topology of network repertoires and inter-group differences, that is, healthy controls ≠ patients with schizophrenia (HC ≠ SCZ), (b) how the integrative importance of nodes changed as a function of task condition, and (c) inter-group similarities and differences in the stability and instability of the observed integrative importance of nodes. In Supporting Information we also report relationships between task proficiency and BC in both groups, and in patients, we report on how well BC was predicted by variables including antipsychotic dosage (Nosé & Barbui, 2008; Nosé et al., 2008), duration of illness, and clinical state (estimated by the Positive and Negative Syndrome Scale (PANSS)) (Kay, Fiszbein, & Opler, 1987). As a complement to existing investigations of resting-state fMRI in SCZ (Alexander-Bloch et al., 2010; van den Heuvel, Mandl, Stam, Kahn, & Hulshoff Pol, 2010), we suggest that this work along with other noted graph theoretic applications to task-based data, accentuates the role of contextual processing in revealing the dynamics of dys-connection in schizophrenia.

## RESULTS

**Figure 1.**
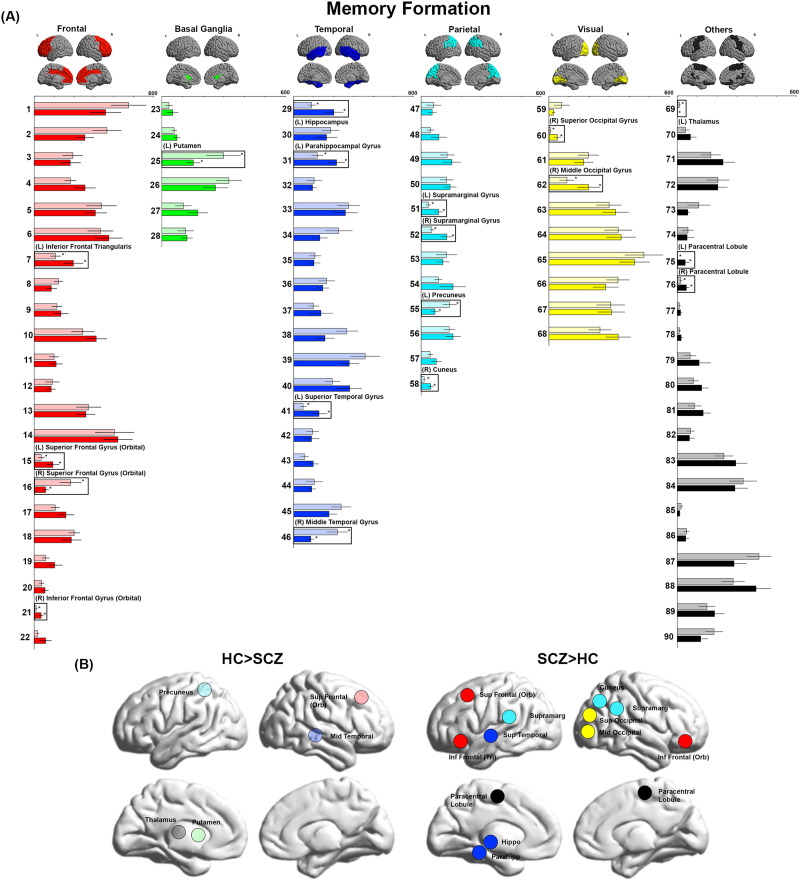
BC during Memory Formation. (A) BC values during Memory Formation are depicted for each of the 90 cerebral parcels (“nodes”). The nodes are organized (and color coded) by lobe (with the color scheme maintained in all subsequent figures). The slightly transparent bars represent healthy controls (HC) and the opaque bars represent patients (SCZ). Significant differences in BC (*p*_FDR_ < 0.05, HC ≠ SCZ), are clearly identified (insets and asterisks). As is evident, Memory Formation induced significant decreases in BC in multiple nodes, including the middle temporal, superior frontal, precuneus, thalamus, and putamen. Conversely, increases in BC were observed in a complement of nodes including the hippocampus, parahippocampal gyrus, superior temporal, superior frontal, inferior frontal, paracentral lobule, supramarginal, cuneus, superior occipital, and middle occipital gyri. (B) For ease of visualization, nodes showing significant inter-group differences are rendered on lateral or medial cortical surfaces where each subfigure denotes nodes with lower BC (left) or higher BC (right) in patients.

**Figure 2.**
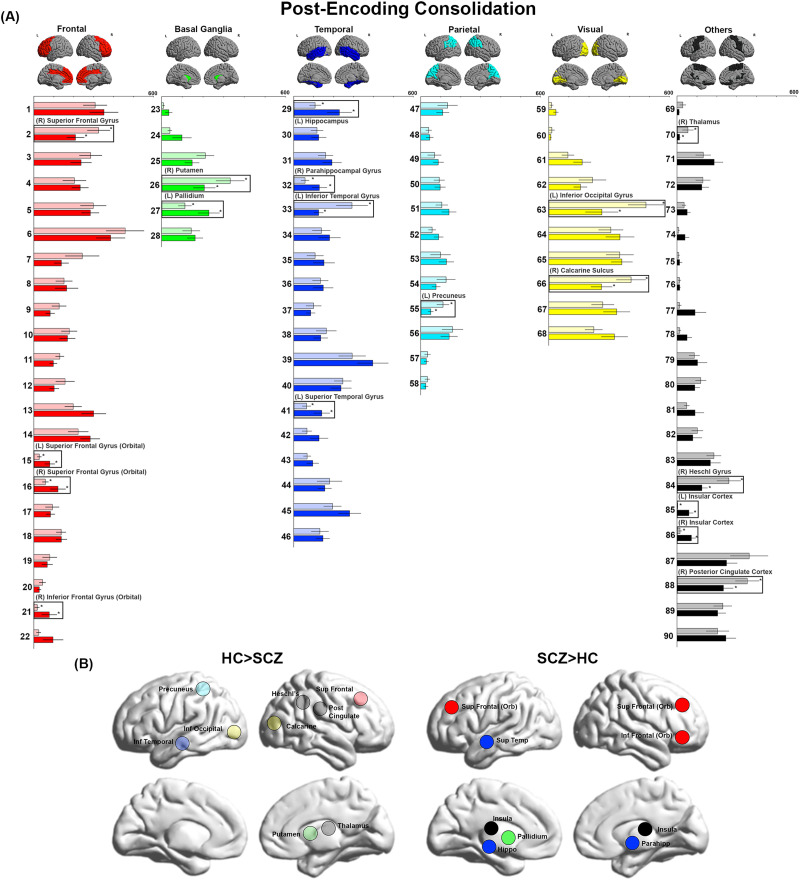
BC during Post-Encoding Consolidation. (A) BC values during Post-Encoding Consolidation are depicted. Even in the absence of sensorimotor stimulation, significant inter-group differences were observed. As seen, we observed decreases in BC in the precuneus, inferior occipital cortex, inferior temporal cortex, calcarine, Heschl’s gyrus, posterior cingulate, superior frontal, putamen, and thalamus. Conversely, we observed increases in BC in the left hippocampus and right parahippocampal gyrus as well as multiple nodes across frontal, temporal, and basal ganglia regions. (B) The locations of nodes with significant inter-group differences are visualized on lateral and medial cortical surfaces.

**Figure 3.**
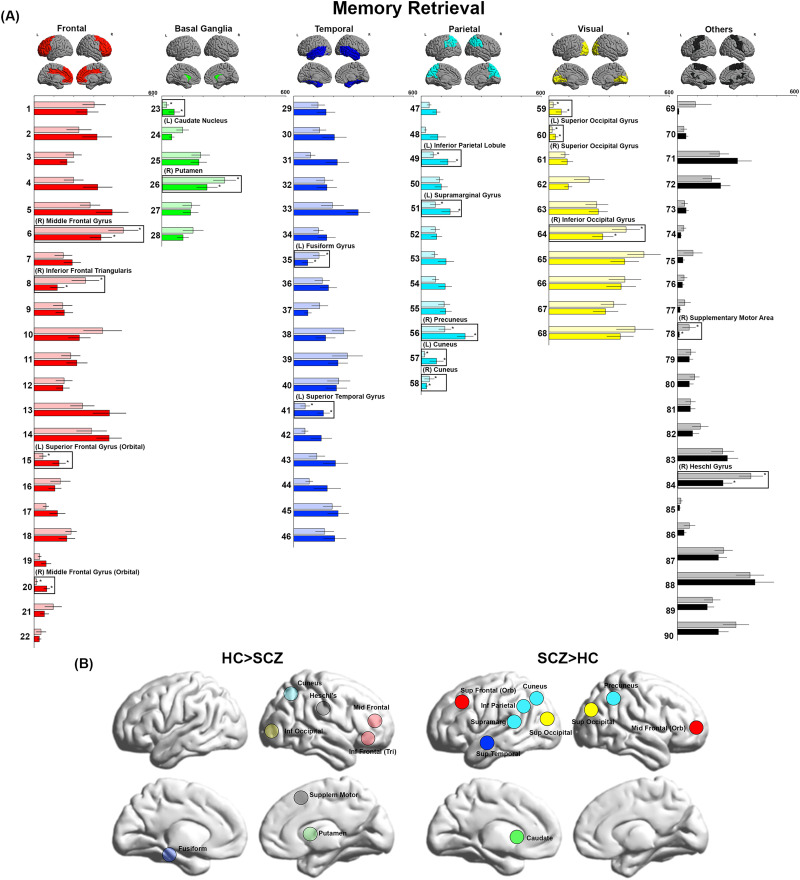
BC during Memory Retrieval. (A) BC values during Memory Retrieval are depicted. We observed decreases in BC in the cuneus, Heschl’s gyrus, mid frontal, inferior frontal, inferior occipital, supplementary motor cortex, putamen, and fusiform. Conversely, we observed increases in BC in the precuneus, superior occipital, mid frontal, cuneus, inferior parietal, supramarginal, superior frontal, superior temporal, and caudate. (B) The locations of nodes with significant inter-group differences are visualized on lateral and medial cortical surfaces.

**Figure 4.**
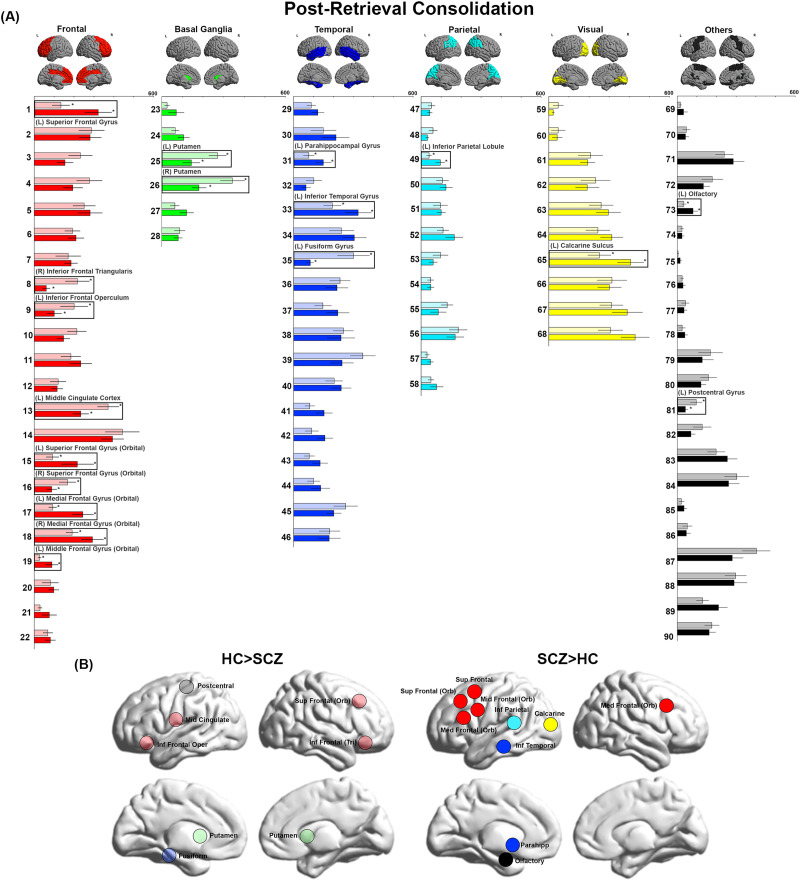
BC during Post-Retrieval Consolidation. (A) BC values during Post-Retrieval Consolidation are depicted. We observed decreases in BC in the postcentral gyrus, fusiform, putamen, and multiple nodes in the frontal lobe. Conversely, we observed increases in BC in the olfactory bulb, as well as multiple frontal, parietal, visual, and temporal lobe nodes. (B) The locations of nodes with significant inter-group differences are visualized on lateral and medial cortical surfaces.

**Figure 5.**
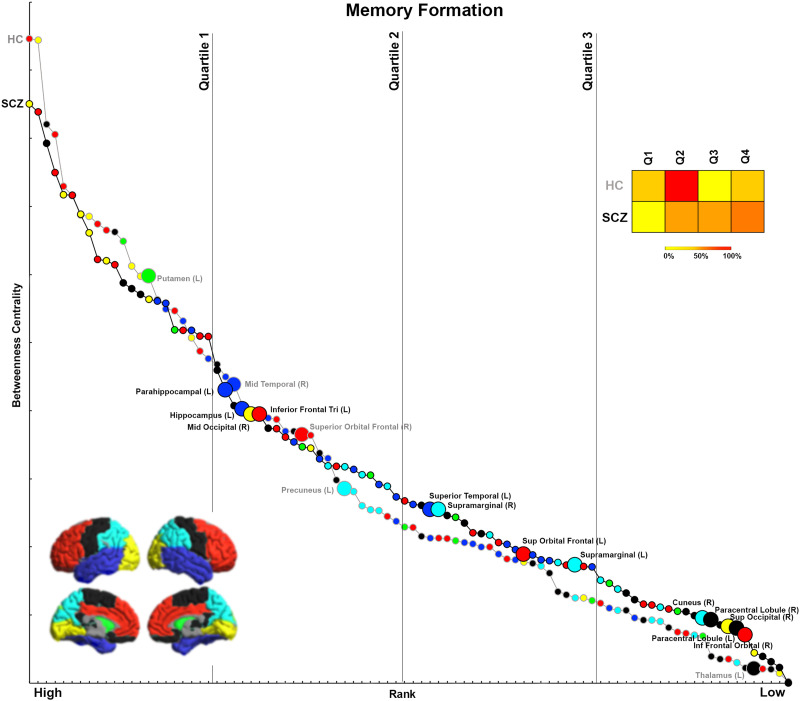
Ranking by BC for Memory Formation. After ranking each node within each group (HC, SCZ) by BC, data from each group are depicted in this two-dimensional space mapping BC (vertical axis) and rank (horizontal axis). Contiguous curves connect nodes within each group thus clearly separating data across HC (gray curve) and SCZ (black curve). Nodes with significant inter-group differences in BC (see Figure 1) are noted (enlarged marker and added node label within the group with the significant increase). Vertical lines on the graph divide the ranking space into quartiles, allowing us to assign nodes with significant differences into rank-based quartiles. The associated heat map represents the percentage of significantly different nodes in each rank-based quartile for each tail of the results (HC > SCZ, SCZ > HC). It is possible to divine the relative importance of significantly different BC measures. For example, as seen in SCZ, nodes with decreased BC tend to be somewhat more highly ranked, but nodes with increased BC tend to be somewhat more lowly ranked.

**Figure 6.**
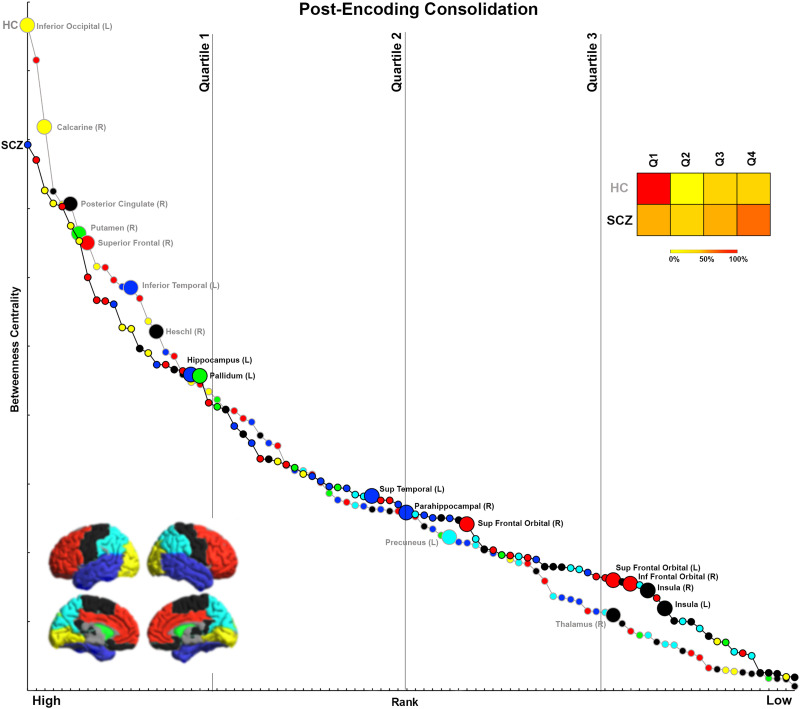
Ranking by BC for Post-Encoding Consolidation. The figure presents data from the Post-Encoding Consolidation condition following the convention of Figure 5. As seen, a high percentage of nodes that have decreased BC in SCZ lie in the first quartile of ranks, indicating that nodes with significantly lower BC in the SCZ group are also highly ranked within the HC network.

**Figure 7.**
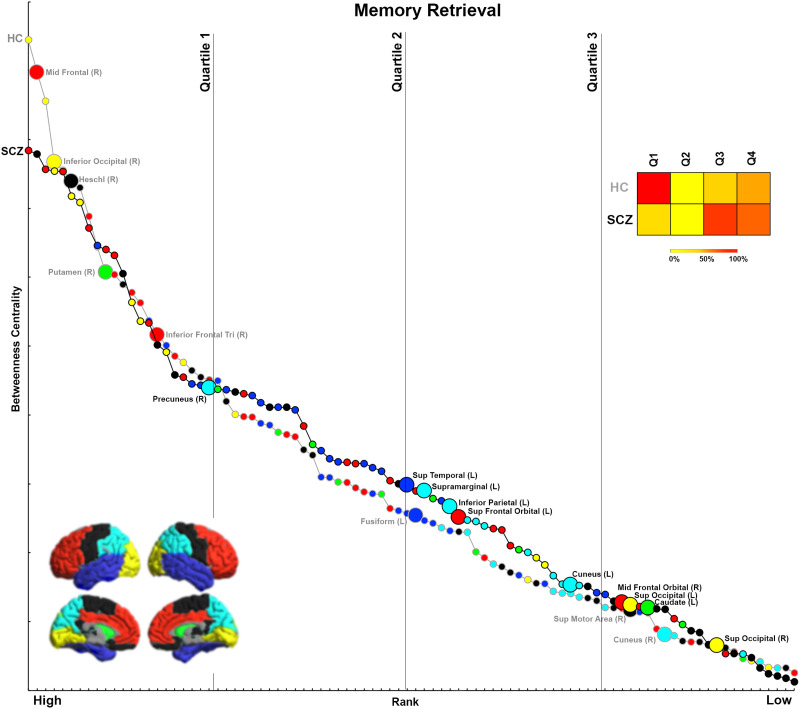
Ranking by BC for Memory Retrieval. The figure presents BC and rank data from each group for the Memory Retrieval condition. Again, nodes with significantly lower BC in SCZ tend to be more highly ranked within the HC network.

**Figure 8.**
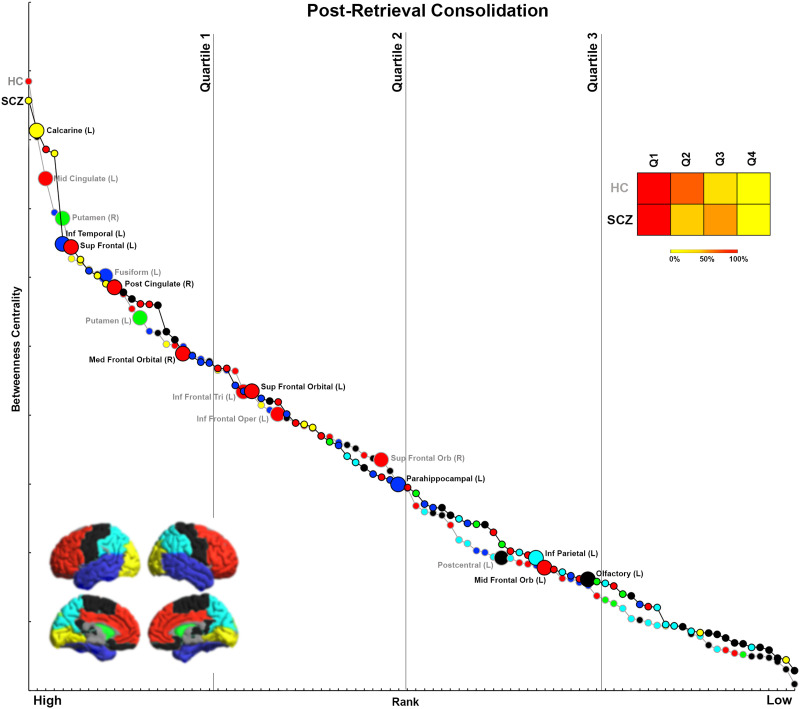
Ranking by BC for Post-Retrieval Consolidation. The figure presents BC and rank data from each group for the Post-Retrieval Consolidation condition. Notably, and unlike in Figures 5–7, nodes with both decreased and increased BC in SCZ tend to be more highly ranked.

**Figure 9.**
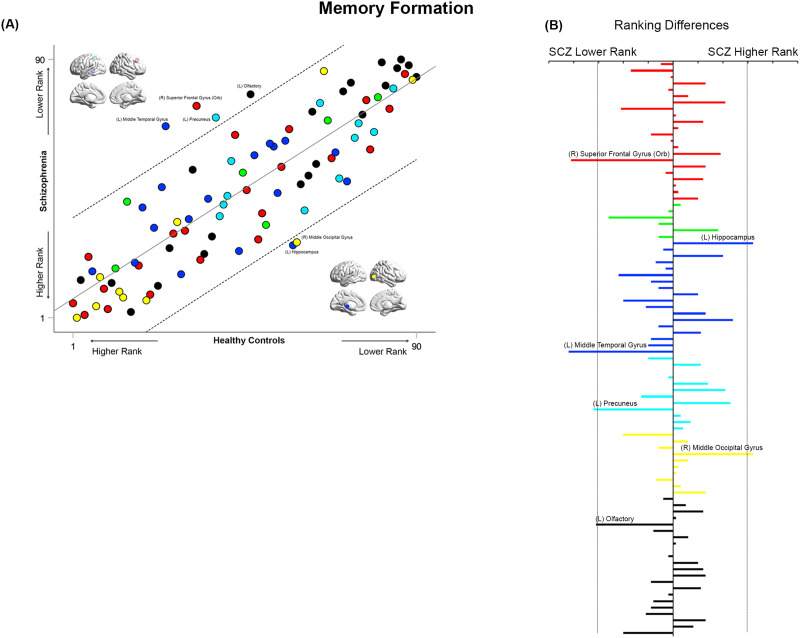
Cross-ranking for Memory Formation. (A) The scatter plot depicts the cross-ranking of nodes across each of the HC (horizontal axis) and SCZ groups (vertical axis). The color coding of nodes is consistent with the general scheme used in the manuscript. The regression line represents the best fit linear model (see Results), with the dashed lines representing the 95% confidence interval for the model. While node ranks are highly correlated across groups, multiple nodes lay outside the confidence interval of the regression model. These include the left middle temporal gyrus, left precuneus, right superior frontal gyrus (orbital), and left olfactory (lower rank in SCZ), and the left hippocampus and right middle occipital gyrus (higher rank in SCZ). (B) To further accentuate the import of the identified nodes from panel A, across all 90 nodes, we computed the absolute inter-group difference in rank. These are depicted in the bar graph (organized from top to bottom along the lines of Supporting Information Table S1). The dropped dashed lines reflect the 95% confidence interval of the model from panel A, and as seen, the absolute value of the difference in rank lies outside the interval for both directions (HC > SCZ and SCZ > HC).

**Figure 10.**
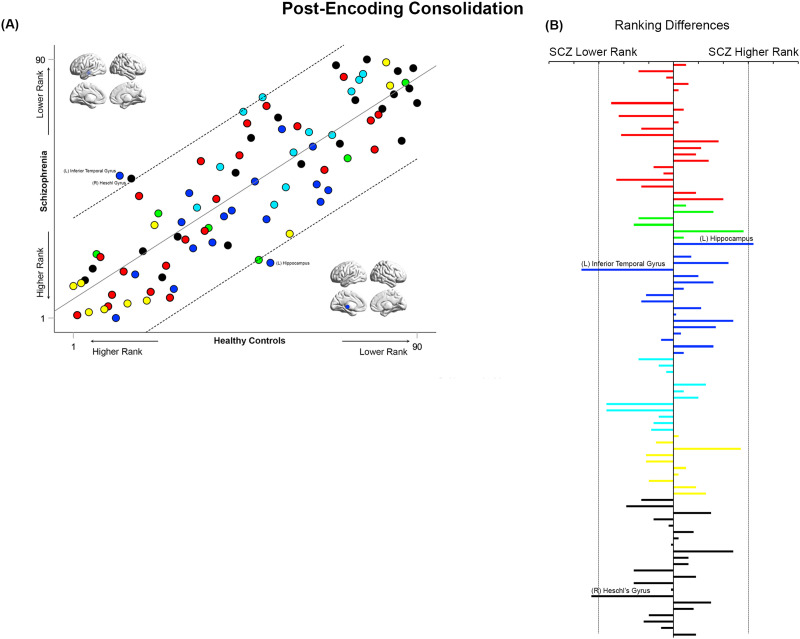
Cross-ranking for Post-Encoding Consolidation. (A) Cross-ranking information (consistent with the format in Figure 9) is presented for the Post-Encoding Consolidation condition. While node ranks are highly correlated across groups, multiple nodes lay outside the confidence interval of the regression model. These include the left inferior temporal gyrus and right Heschl’s gyrus (lower rank in SCZ), and the left hippocampus (higher rank in SCZ). (B) The absolute inter-group difference in rank is presented in the same convention as Figure 9.

**Figure 11.**
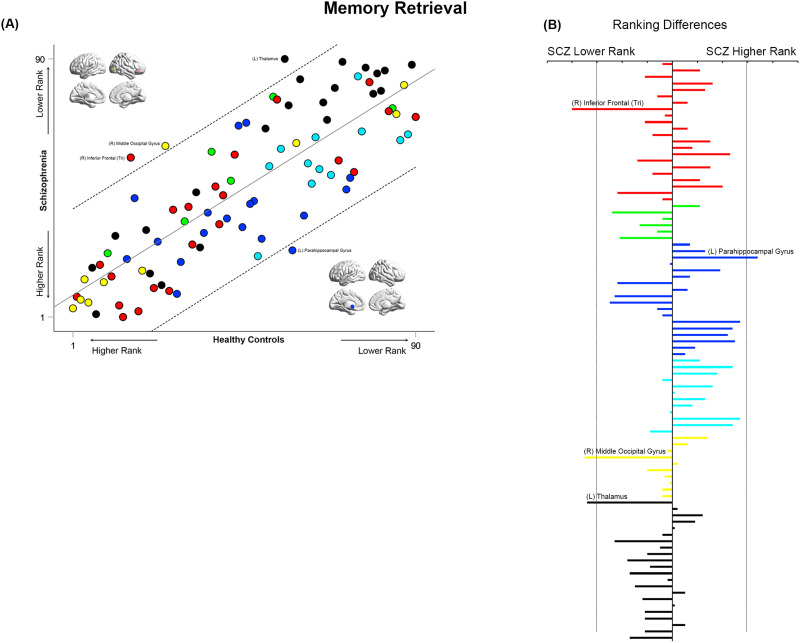
Cross-ranking for Memory Retrieval. (A) Cross-ranking information (consistent with the format in Figures 9–10) is presented for the Memory Retrieval condition. While node ranks are highly correlated across groups, multiple nodes lay outside the confidence interval of the regression model. These include the right inferior frontal triangularis, right middle occipital gyrus, and left thalamus (lower rank in SCZ), and the left parahippocampal gyrus (higher rank in SCZ). (B) The absolute inter-group difference in rank is presented in the same convention as Figures 9–10.

**Figure 12.**
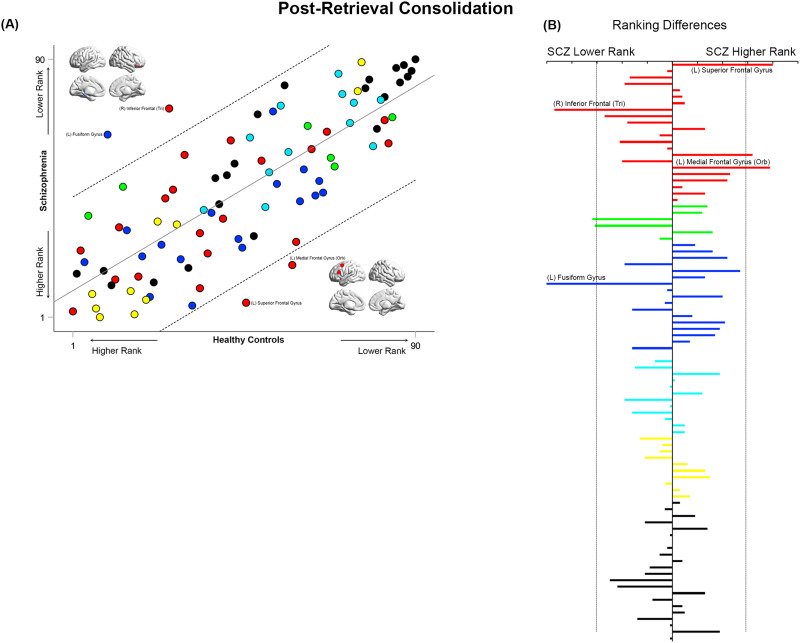
Cross-ranking for Post-Retrieval Consolidation. (A) Cros- ranking information (consistent with the format in Figures 9–11) is presented for the Post-Retrieval Consolidation condition. While node ranks are highly correlated across groups, multiple nodes lay outside the confidence interval of the regression model. These include the left fusiform gyrus and right inferior frontal triangularis (lower rank in SCZ), and the left superior frontal and medial frontal orbital (higher rank in SCZ). (B) The absolute inter-group difference in rank is presented in the same convention as Figures 9–11.

**Figure 13.**
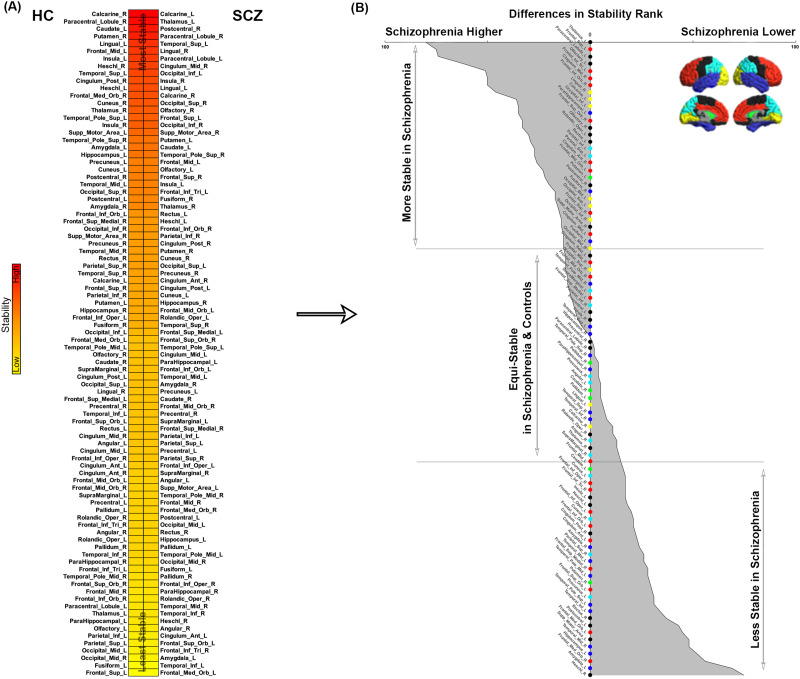
Network stabilities. (A) In each group, nodes are arranged in descending order of the stability of their rank across conditions. As seen, across groups, the ordering is different suggesting that the stability of node ranks varied greatly by node. (B) To quantify this, across all 90 nodes, we calculated the difference in rank by stability (SCZ minus HC). Resultant values were reordered from most negative (most stable in schizophrenia) to most positive (least stable in schizophrenia). We plotted the 90 nodes and shaded the area under the curve to reflect the difference in rank by stability between SCZ and HC (naturally, the relative stability map presents symmetrically). In addition to listing node identity, for ease of access we also code the vertical access with colors reflecting lobe assignments for each node (see lateral and medial brain representations). Then, the 90-node space was cleaved (horizontal lines) into tertiles representing in sequence, (a) nodes more stable in SCZ, (b) nodes that were relatively equi-stable, and (c) nodes less stable in SCZ.

**Figure 14.**
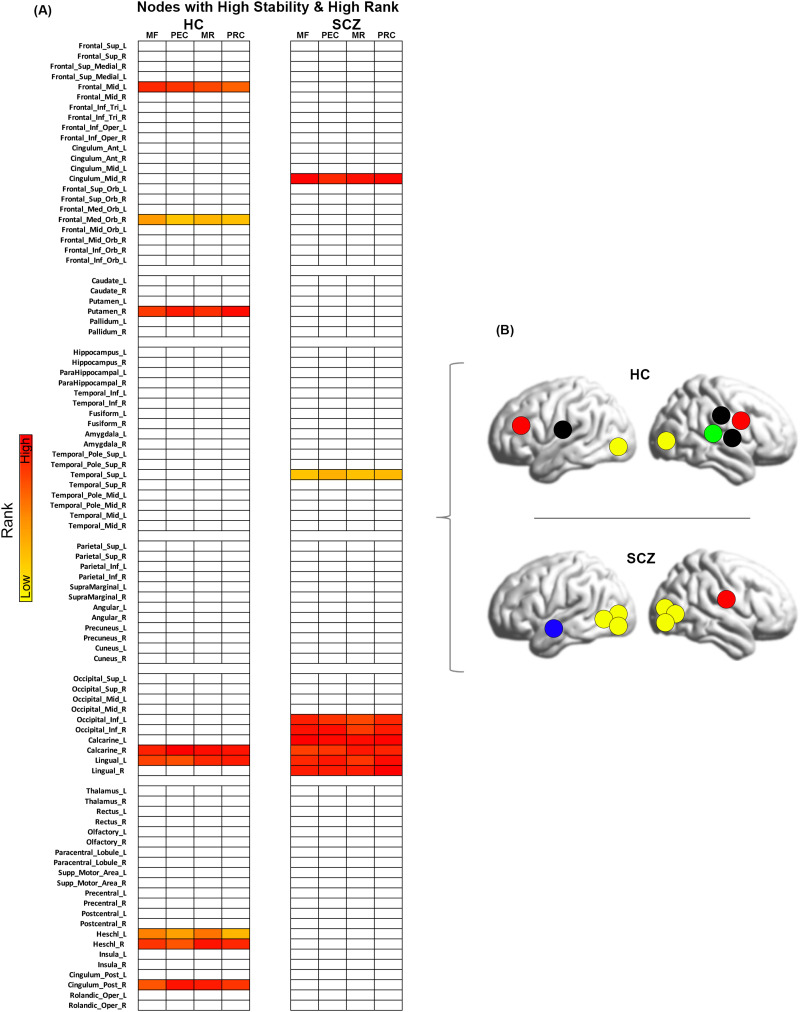
Nodes with high stability and high rank. (A) The figure depicts the nodes that are both highly stable and highly ranked (across the conditions of Memory Formation (MF), Post-Encoding Consolidation (PEC), Memory Retrieval (MR), and Post-Retrieval Consolidation (PRC)) in each of the HC and SCZ groups. These nodes are particularly salient to our analyses because they underpin network implementation independent of task condition. (B) Lateral brain representations of the network topology (obtained from panel A) for both HC and SCZ are depicted here. As can be clearly seen, the network stability varies between groups, with SCZ evincing a highly circumscribed organization of nodes and HC displaying a more distributed network.

We organize the presentation of results in the following logical order. First, we first provide a comprehensive accounting of inter-group differences in BC across each of the four experimental conditions (Figure 1A, Figure 2A, Figure 3A, and Figure 4A). For ease of access, nodes are classified by lobe (frontal, basal ganglia, temporal, parietal, visual, others; Supporting Information Table S1 provides a listing of regional classifications). Next, in Figure 5, Figure 6, Figure 7, and Figure 8, which are based on rank ordering by BC, we examine the relative intranetwork importance of nodes. Third, these emerging results led to further explorations of the relationship between node rankings across groups in each of the four conditions (Figure 9A, Figure 10A, Figure 11A, and Figure 12A). Fourth, in a final assessment, in each group, we identified nodes with stable and high ranks across all four memory conditions (Figure 13 and Figure 14).

### Betweenness Centrality Analysis

In Figures 1A–4A, the bar graphs represent the mean (±*SEM*) BC for each group for that node (SCZ are represented by opaque colors, whereas HC are represented by transparent colors). Nodes with significant inter-group differences (*p*_FDR_ < 0.05, SCZ ≠ HC) are identified by insets (and labels). In Figures 1B–4B, nodes with significant inter-group differences are presented on lateral or medial cortical surfaces (for a succinct visual summary).

### Memory Formation

As seen, in SCZ significant reductions in BC were observed in the right superior frontal gyrus (orbital), the left putamen, the right middle temporal gyrus, the left precuneus, and the left thalamus. Conversely, increased BC was observed across several regions in the frontal lobe (left inferior frontal triangularis, left superior frontal gyrus (orbital), right inferior frontal gyrus (orbital)), the temporal and medial temporal lobe (left hippocampus, left parahippocampal gyrus, left superior temporal gyrus), the parietal lobe (bilateral supramarginal gyrus, right cuneus), and the visual lobe (right superior occipital gyrus, right middle occipital gyrus).

### Post-Encoding Consolidation

SCZ showed decreased BC across several lobes, including the frontal (right superior frontal gyrus), temporal (left inferior temporal gyrus), parietal (left precuneus), and visual cortices (left inferior occipital gyrus and right calcarine sulcus), and the basal ganglia (right putamen). Decreases were also observed in the right thalamus, right Heschl’s gyrus, and right posterior cingulate cortex. Conversely, we observed increases in BC in multiple lobes, including the frontal (bilateral superior frontal gyrus (orbital) and the right inferior frontal gyrus (orbital)), basal ganglia (left pallidum), and temporal (left hippocampus, right parahippocampal gyrus, left superior temporal gyrus) as well as the bilateral insular cortex.

### Memory Retrieval

Decreased BC was observed in eight nodes across the frontal lobe (right middle frontal gyrus, right inferior frontal triangularis), basal ganglia (right putamen), temporal lobe (left fusiform gyrus), parietal lobe (right cuneus), and visual cortex (right inferior occipital gyrus). Additionally, decreased BC was observed in the right supplementary motor area and right Heschl’s gyrus. By comparison, increased BC was observed in 10 nodes, centered mostly around the parietal lobe (left inferior parietal lobule, left supramarginal gyrus, right precuneus, left cuneus), with additional effects in the frontal lobe (left superior frontal gyrus (orbital) and the right middle frontal gyrus (orbital)), basal ganglia (left caudate nucleus), temporal lobe (left superior temporal gyrus), and visual cortex (bilateral superior occipital gyrus).

### Post-Retrieval Consolidation

Decreased BC was observed primarily in frontal nodes (right inferior frontal triangularis, left inferior frontal operculum, left middle cingulate cortex, and right superior frontal gyrus (orbital)). Moreover, decreases were also observed in the basal ganglia (bilateral putamen), temporal lobe (left fusiform gyrus), and left postcentral gyrus. Increased BC was observed in a complement of nodes in the frontal lobe (left superior frontal gyrus, left superior frontal gyrus (orbital), bilateral medial frontal gyrus, and left middle frontal gyrus (orbital)). Increased BC was also noted in the temporal lobe (left parahippocampal gyrus and left inferior temporal gyrus), parietal lobe (left inferior parietal lobule), visual cortex (left calcarine sulcus), and the left olfactory bulb.

Observed effect sizes (Cohen’s *d*) were estimated across all analyses presented in Figures 1–4, to assess any limiting role of the employed sample size (*n* = 59) on the observed inter-group differences. A wide range of effect sizes were observed (.001 ≤ Cohen’s *d* ≤ .83). Notably, 55% of the values lay in the small to medium, or higher range (Cohen’s *d* ≥ .2), with 14% lying in the medium to large range (Cohen’s *d* ≥ .5) (Cohen, 1988).

In further explorations from these main results (Supporting Information Figures S6 and S7), (a) relationships between behavioral performance and BC (across and within groups), and (b) within SCZ, relationships between clinical variables and antipsychotic dosage, and BC were explored using correlational analyses. These analyses were conducted node-wise in each of the four conditions. We observed sparse convergence between nodes with significant relationships in these exploratory analyses and nodes with significant inter-groups differences (Figures 1–4) but more focused independent investigations are needed.

### Analyses of Rankings Based on BC

Initial analyses (Figures 1–4) identified nodes with significant inter-group differences. However, these results are agnostic about the integrative importance of these nodes within each participant group and condition. To investigate whether nodes with different BC were more (or less) important within their network, BC was used to rank nodes in each group and condition. This ranking is an ordinal measure of the integrative importance of any node. For each condition (Figure 5–8), the nodes are arranged in order of rank, separately for each group. Curves are used to distinguish between SCZ (black line) and HC data (gray line), with color coding for the lobes maintained. Only nodes with significant differences (from Figures 1–4) are labeled (and enlarged for visual access). To simplify appraisal, the space is divided by vertical lines. These divide the ranking space into quartiles (based on ranking). Finally, heat maps represent the percentage of significantly different nodes for each direction (HC > SCZ, SCZ > HC) that fall within each of the four quartiles, and they permit assessment of the relative importance of nodes with significant differences.

### Memory Formation

In SCZ, 80% of nodes with significantly lower BC (relative to HC) were highly ranked (i.e., in the first and second quartiles) within the respective networks. These included the putamen, middle temporal, superior frontal orbital, and precuneus. Conversely, 70% of the nodes with significantly higher BC (relative to HC) were lowly ranked (i.e., in the third or fourth quartiles) within the network, including nodes such as the inferior frontal orbital, paracentral lobule, superior occipital lobule, and cuneus.

### Post-Encoding Consolidation

As with Memory Formation, 80% of nodes with significantly lower BC in SCZ were highly ranked (seen solely in the first quartile of ranks), including the inferior occipital, calcarine, posterior cingulate, putamen, superior frontal, inferior temporal, and Heschl’s gyrus. Conversely, a relatively large percentage of nodes (∼65%) with significantly higher BC were lowly ranked, including the parahippocampal gyrus, superior frontal orbital, inferior frontal orbital, and insula.

### Memory Retrieval

In SCZ, ∼65% of nodes with significantly lower BC were highly ranked (in the first quartile), including the middle frontal, inferior occipital, Heschl’s gyrus, putamen, and inferior frontal triangularis. Conversely, 90% of nodes with significantly higher BC were lowly ranked (i.e., in the third and fourth quartiles) within the network. These include the superior temporal, supramarginal gyrus, inferior parietal, superior frontal orbital, cuneus, middle frontal orbital, superior occipital, and caudate.

### Post-Retrieval Consolidation

Only during Post-Retrieval Consolidation was a distinct trend observed. Here, most nodes with decreases in BC (90%) were highly ranked. These included the middle cingulate, putamen, fusiform, inferior frontal triangularis and operculum, and superior frontal orbital. Moreover, 70% of nodes with increases in BC were also highly ranked. These included the calcarine, inferior temporal, superior frontal, posterior cingulate, medial frontal orbital, superior frontal orbital, and parahippocampal gyrus.

### Inter-group Similarities in General Network Topology Based on Node Ranks

How similar are the groups in terms of “general” network topology features? We operationalized this question by investigating the inter-group relationships between node rankings. These rankings provide an ordinal measure of the relative integrative importance of a node within that group (and condition). Therefore, quantifying the correlations of ranks provide a measure of inter-group consistency in general network topology. High cross-correlations would suggest that rank order is largely maintained, and that general network topology is more similar than different between groups. In such case, it would be particularly important to identify nodes that are outliers in each correlation model. These questions were investigated using linear regression models applied to the observed ranks of the 90 nodes in each of the SCZ and HC groups in each of the four conditions (see Figures 9A–12A). Outliers in the regression models lying outside the 95% confidence interval of each model are noted for differences in ranking.

Across all conditions, node ranks were highly correlated across groups (all *r*^2^ ≥ 0.63, *F*_1,88_ ≥ 151.6), suggesting that general network topology was more similar than different. However, in each of the four experimental conditions, specific nodes lay outside the 95% confidence interval of the model. In each of Figures 9B–12B, the bar graphs represent the absolute difference in observed rank between groups (the dropped lines represent the 95% confidence interval in each model).

During Memory Formation (Figure 9), the middle temporal gyrus, superior frontal gyrus (orbital), precuneus, and olfactory bulb all lay outside the confidence interval, suggesting a substantially lower ranking in SCZ. By comparison, the hippocampus and middle occipital gyrus lay outside the confidence interval, suggesting a substantially higher ranking.

During Post-Encoding Consolidation (Figure 10), the inferior temporal gyrus and Heschl’s gyrus appeared to have a substantially lower ranking in SCZ, whereas the hippocampus had a higher ranking.

During Memory Retrieval (Figure 11), the inferior frontal triangularis, middle occipital gyrus, and thalamus had a lower ranking in SCZ, whereas the parahippocampal gyrus had a higher ranking.

Finally, during Post-Retrieval Consolidation (Figure 12), the fusiform gyrus and inferior frontal triangularis had a lower ranking in SCZ, whereas the superior and medial frontal gyri had a higher ranking.

### Stable and Unstable Ranks Across Conditions

Within any condition and group, a node’s ranking (based on BC) reflects its relative importance to the functional organization of the network. How stable is this relative importance across conditions? Addressing this issue allows us to capture some measure of the stability and instability of network repertoires across task conditions in each of the SCZ and HC groups.

To estimate this, we first calculated the standard deviation (*SD*) of each node’s ranking across conditions, where a low SD represents a high degree of stability in the node’s rank. In Figure 13A, for each of the HC and SCZ groups, nodes are presented in descending order of stability, showing that some nodes (e.g., the right hippocampus) are equi-stable, whereas other nodes (e.g., the left hippocampus) show greatly differing stabilities across groups. Across all 90 nodes, we next created a relative stability map (Figure 13B) with values (absolute difference in the stability rank) representing the difference in inter-group stability of that node. For ease of access we code the markers on the vertical axis to reflect lobe assignments (while also listing node identity). Then, the 90-node space was cleaved (horizontal lines) into tertiles representing in sequence, (a) nodes *more* stable in SCZ, (b) relatively equi-stable nodes and, (c) nodes *less* stable in SCZ.

Salient to our interests were nodes that were both highly ranked and highly stable. These nodes could reasonably be thought to underpin network organization independent of task condition (therefore generally being more “hub” like). Of the 16% of nodes with the lowest *SD* (i.e., the most stable in terms of rank), Figure 14 represents those which showed the *highest* rank (∼9% of the total nodes). Thus, the figure represents highly stable *and* highly ranked nodes in each group. As seen, in HC, these cross-condition “hubs” were distributed across the cortex and included nodes in the frontal lobe (mid frontal, medial frontal orbital), the putamen, the bilateral Heschl’s gyrus, the posterior cingulate, and nodes in the visual lobe (calcarine and lingual). In notable contrast, the highly circumscribed locations of the stable hubs in schizophrenia primarily lay in the visual lobe (bilateral inferior occipital, bilateral calcarine, and bilateral lingual gyrus), except for the mid cingulate and superior temporal.

## DISCUSSION

Using an associative learning paradigm with multiple conditions, we induced brain network dynamics in SCZ patients and controls to (a) characterize resultant network repertoires based on betweenness centrality (BC) (Z. Chen & Calhoun, 2018; Cheng et al., 2015; Lord, Horn, Breakspear, & Walter, 2012), (b) examine group (HC ≠ SCZ) differences in estimated BC, (c) assess the relative importance (based on rank ordering by BC) of significantly different nodes within each group’s network, (d) examine the relationship between the ranks of nodes across groups, and (e) assess the relative stability of the node rankings across conditions. These investigations revealed four salient results: (a) inter-group differences in BC were observed across experimental conditions (even during periods of passive memory consolidation) (Figures 1–4); (b) nodes with increased BC in SCZ were lowly ranked, whereas nodes with decreased BC in SCZ were highly ranked (Figures 5–8); (c) in each of the conditions, observed ranks were correlated across groups (Figures 9–12), but differed for several task-relevant nodes; (d) finally, in SCZ, nodes with high rank stability were isolated to early sensory cortex, but in HC, were distributed across the cerebral cortex (Figures 13–14).

Previous studies of schizophrenia have characterized network topology of resting-state fMRI data (Cheng et al., 2015; Nijhuis, van Cappellen van Walsum, & Norris, 2013; Oldham et al., 2019), but increasingly, studies are now attempting to understand the impact of task-driven contextual processing on disordered functional network topology in schizophrenia. This task-driven approach is valuable because fMRI signals are maximally responsive to task-driven modulation (Logothetis, 2008), and tasks amplify expressions of clinical pathology in networks (Diwadkar & Eickhoff, 2021a). We specifically relied on BC because it can be estimated from undirected graphs (as in our case) (Dablander & Hinne, 2019), and combines both degree and path length in its estimation (Bullmore & Sporns, 2009; Zuo et al., 2012). Finally, in estimating the integrative properties of nodes, BC is particularly well suited for characterizing a dys-connection syndrome like schizophrenia (Medaglia, Lynall, & Bassett, 2015). In the remainder of the Discussion, we unpack the import of the results, initially focusing on group differences within each task condition, before discussing observed stability and instability of repertoires across task conditions.

### Inter-group Differences and Node Rankings

Memory formation is a dynamic process of sensing or perceiving novel information or memoranda, that are initiated into the preliminary process of learned associations (Bero et al., 2014). It has widely distributed cerebral correlates, with nodes in the medial temporal lobe, the prefrontal cortex, the striatum, and the thalamus playing highly integrative roles (Pardi et al., 2020; Squire, Stark, & Clark, 2004). While we observed an admixture of differences in inter-group effects (SCZ ≠ HC) (Figure 1B), many nodes with increased BC in SCZ were relatively lowly ranked (in the third and fourth quartiles; see Figure 5), suggesting that nodes with increased integrative roles were less important. Two notable exceptions were the hippocampus and parahippocampal gyrus. All inter-group differences are aberrant, so if the integrative role of these nodes is greater in patients, why might such a finding result? “Compensatory” increases in fMRI responses typify the schizophrenia literature (Kim et al., 2010) and may reflect inefficient rather than adaptive responses (such as those seen in normal aging) (Reuter-Lorenz & Cappell, 2008). Indeed, increased hippocampal activity has been observed both at rest (Tregellas et al., 2014) and in tasks ranging from learning (Wadehra, Pruitt, Murphy, & Diwadkar, 2013) to sensory gating (Tregellas et al., 2007). As Supporting Information Figure S1 shows, patient performance lagged that of their healthy counterparts, reinforcing this inference.

Effortful Memory Retrieval is initiated by cues from the prefrontal cortex (Simons & Spiers, 2003; Woodcock, White, & Diwadkar, 2015), supplemented by the dorsal striatum. The retrieval trace interjects with distributed memory representations in regions including the hippocampus (Kragel & Polyn, 2015). Accordingly, the significant decrease in BC in the middle frontal gyrus (along with the inferior frontal triangularis, the inferior occipital gyrus, Heschl’s gyrus, and the putamen) (Figure 3) suggests a decreased integrative role during retrieval. Moreover, these nodes were highly ranked, confirming that schizophrenia is compromised by a loss of the integrative role of regions in the prefrontal cortex that underpins the successful retrieval of memory traces (Takehara-Nishiuchi, 2021).

A notable observation was that disordered network repertoires in schizophrenia were evoked even during periods free of overt sensorimotor stimulation or processing (Figures 2, 4, 6, and 8). Rest states within task-driven studies have ongoing and active processes that are related to the foreground tasks that these states are a part of (Diwadkar, Asemi, Burgess, Chowdury, & Bressler, 2017; Ravishankar et al., 2019). However, as is known, these covert repertoires evince unique properties that are inherited from task-active states.

Consolidation drives the recapitulation of as-yet weak memory traces through covert rehearsal (Lilienthal, Myerson, Abrams, & Hale, 2018) and/or spontaneous brain network reorganization (Malerba & Bazhenov, 2019). Two aspects of our observed group differences are notable. First, although both consolidation conditions were superficially identical, the evoked disordered network repertoires were different (Figures 2B vs. Figure 4B). During Post-Encoding Consolidation, reduced BC was observed in highly ranked nodes, but increased BC was primarily seen in lowly ranked nodes. However, during Post-Retrieval Consolidation nodes with inter-group differences (Figure 4) were highly ranked (Figure 8), and the locations of these nodes were (by definition) complementary. In SCZ, the integrative role of multiple nodes in the frontal lobe was higher, suggesting an exaggerated role in attempting to recapitulate and integrate inadequately formed memory traces that rely on transient working memory (He et al., 2012; Yang et al., 2020). Second, disordered repertoires during recapitulation conditions were only partially overlapping with those observed during preceding task-active conditions (Figures 1B and 3B, respectively), further evidence that constructive covert psychological processes drive unique network repertoires (Diwadkar et al., 2017; Jacobacci et al., 2020; Ravishankar et al., 2019).

### Cross-Rankings: Similarities and Differences in Network Repertoires

Analyzing cross-rankings of individual nodes (Figures 9–12) provides a window into consistencies and differences in network repertoires observed in each condition. Node ranks were highly correlated across groups in each of the conditions. This consistency indicates that even highly debilitating conditions like schizophrenia are marked by only subtle differences in network repertoires (Lam, Li, Ke, & Yung, 2022; Sprooten et al., 2017), This subtlety may arise because (a) unlike long-standing neurologic conditions, neuropsychiatric diseases affect brain function at its “margins” (Spronk et al., 2021); and (b) thus any observed impacts are contextually evoked (Robison, Thakkar, & Diwadkar, 2020). Nevertheless, in these analyses, the hippocampus and parahippocampal gyrus showed higher ranks in patients during Memory Formation and Retrieval, but nodes such as the superior frontal gyrus and the inferior frontal triangularis showed lower ranks. These effects reemphasize the exaggerated centrality of medial temporal lobe structures in schizophrenia during active task conditions, and they highlight the dynamic nature of inter-group differences in network repertoires as driven by task context. We note that an overwhelming focus on positive results (i.e., significant inter-group differences), frequently obscures appreciation of the subtle nature of effects, even in conditions as debilitating as schizophrenia (Bouttier, Duttagupta, Denève, & Jardri, 2022).

### The Stability of Node Ranks Across Conditions

The final analyses revealed transitions in response to changing task conditions (Najafi, McMenamin, Simon, & Pessoa, 2016). When ordered by stability of ranks (Figure 13), equal numbers of (complimentary) nodes in the prefrontal cortex are more or less stable in patients (Figure 13B). More salient is evidence (Figure 14) that in healthy controls, highly stable and highly ranked nodes are distributed across the cortex (Mattar, Cole, Thompson-Schill, & Bassett, 2015). This finding is further evidence that while stable and flexible repertoires are a highlight of functional adaptation and efficiency in the healthy brain (Liu, Kohn, & Fernandez, 2021; Morin, Chang, Ma, McGuire, & Stern, 2021; Sporns & Kotter, 2004), this adaptability is implemented at alternative sites in schizophrenia.

### Limitations and Conclusions

Capturing network repertoires from an inherently dynamical system like the human brain is challenging (Park & Friston, 2013), yet the use of well-titrated tasks coupled with the application of graph theoretic measures like BC can be enormously useful (Rubinov & Sporns, 2010). In addition to assessing differences in BC (as we initially did), the measure provides an ordinal representation of the integrative importance (“hubness”) of nodes in a network. From a rich set of analytic targets, we arrived at several insights. Generally, cerebral nodes with higher BC in patients were more lowly ranked (with the opposite being true in healthy controls). This observation suggests that task implementation drives alternative network topology in schizophrenia. Remarkably, passive memory consolidation drove altered network topology even in the absence of overt stimulation or processing. While some aspects of network topology (based on the cross-ranking of nodes) was similar across groups, meaningful exceptions emerged. And finally, in controls a cross-cerebral network of nodes showed high rank stability and high ranking, but in SCZ, this similarly classified network was highly localized to early sensory regions. Such localization is suggestive of an altered reliance on sensorial as opposed to integrative processing in the illness.

Are the observed effects specific to schizophrenia, and if not, how likely are they to be observed across other conditions with disordered long-term memorial processing? Impairments in learning and episodic memory cut across disorders of mood, anxiety, and personality (Brambilla et al., 2011; Carcone, Lee, & Ruocco, 2020; Quraishi & Frangou, 2002; Ruocco & Bahl, 2014; Sauro, Jorgensen, & Pedlow, 2003), though similarities and/or differences in network repertoires have not been systematically addressed. As with other studies in this domain, a lack of a clinical control group limits our ability to draw definitive conclusions about the diagnostic specificity of our results. On a separate point of note, we also cannot definitively conclude that disordered network topology in schizophrenia is tied to the specific task used. Generally, brain network repertoires reflect task-evoked profiles, one reason for why structural brain network connectivity underpredicts functional connectivity (Park & Friston, 2013). Indeed, the contextually (i.e., condition) driven bases of our results suggest that schizophrenia may be better characterized as a syndrome with a propensity for task-evoked dys-connection, rather than simply as a dys-connection syndrome. However, within-participant comparisons using a multiplicity of datasets would be needed to reach such conclusions. Across nodes, BC estimates (regardless of task condition), were not particularly well predicted by behavioral performance (Supporting Information Figure S6) or medication dosage (patients) (Supporting Information Figure S7). This sparsity suggests that estimates of the integrative value of nodes were uncoupled from participant’s task proficiency or “how” medicated patients were. These interrelationships will probably need to be investigated in more focused and systematic analyses. Finally, our estimates of effect size suggest that our sample size was viable for identifying inter-group differences, our sample was admittedly smaller than several other task-based studies in schizophrenia (and certainly smaller than resting-state studies).

The road toward long-term memory consolidation is “long and winding” (Eichenbaum, 2001). Because such consolidation is undeniably compromised in schizophrenia, learning and memory is the subject of multiple intervention strategies (Manoach, Mylonas, & Baxter, 2020). Laboratory tasks are of limited ecological validity in addressing such fundamental questions. However, concurrently with functional neuroimaging, such tasks can be deployed to provide insights into disordered network repertoires that underlie associative learning and memory in schizophrenia. More than activation-based measures, complex graph theoretic metrics that summarize contextually driven network repertoires may be more useful biomarkers of the illness and/or of treatment efficacy (Diwadkar & Eickhoff, 2021b).

## METHODS

### Participants

Wayne State University’s institutional review board approved all procedures. We collected functional-MRI (fMRI) data from 59 participants (32 stable SCZ, 39 male, 20 female; mean age: HC, 28.02 ± 6.69; SCZ, 29.98 ± 8.38) recruited from the greater Detroit area through local advertisements. Participants provided informed consent and subsequently received remuneration for their involvement. SCZ patients were identified through their treating physicians, and the diagnosis was confirmed by a research psychologist using DSM-V criteria for SCZ (American Psychiatric Association, 2013). All patients were maintained on a regimen of atypical antipsychotics (risperidone, olanzapine, or aripiprazole). Clinical symptom severity ratings were assessed using the PANSS (Kay et al., 1987). General intelligence was assessed using the Wechsler Abbreviated Scale of Intelligence (Psychological Corporation, 1999). The duration of illness was estimate from the most likely date of onset of psychotic symptoms (hallucinations, delusions, or disorganization of thinking; bizarre or catatonic behavior) and date of diagnosis for SCZ, using all available information (medical records, reports by family members or significant others, and the Structured Clinical Interview for DSM Disorders interview). HC participants were free of Axis-I psychopathology (past/present). Participants were screened prior to entering the study to exclude any significant past/current medical and/or neurological illness (e.g., hypertension, thyroid disease, diabetes, asthma requiring prophylaxis, seizures, or significant head injury with loss of consciousness). The two groups did not differ in age or gender distribution. Demographic data, clinical characterization, and medication lists (patients) are shown in Table 1.

**Table 1.** Demographics, medication, and clinical characteristics

	SCZ (*n* = 32)	HC (*n* = 27)
Demographics		
Age (years)	29.98 ± 8.4	28.02 ± 6.7
Sex (% female)	10 (31%)	10 (37%)
IQ	89.9 ± 10.7	91.9 ± 13.0
Clinical information		
Duration of illness (years)	7.8 ± 6.1	
PANSS	51.2 ± 9.7	

*Note*. The table provides demographic and clinical information for the group of 59 participants. All patients were stabilized on a regime of antipsychotics. In addition, two (6%) were prescribed antidepressants, eight (25%) anxiolytics, and seven (22%) mood stabilizers. Clinical symptom severity was assessed using the Positive and Negative Syndrome Scale (PANSS) (Kay et al., 1987) (total PANSS is reported). The duration of illness was derived from the most likely date of onset of psychotic symptoms (hallucinations, delusions, or disorganization of thinking; bizarre or catatonic behavior) using all clinical information, including medical records, reports by family members or significant others, and the Structured Clinical Interview for DSM Disorders interview.

### MRI Acquisition

Data (3 T Siemens Verio scanner, 32-channel volume head coil) were acquired using a multiband gradient EPI sequence (TR = 3 s, TE = 24.6 s, multiband factor = 3, FOV = 192 × 192 mm^2^, matrix = 96 × 96, 64 axial slices, resolution = 2 mm^3^). T_1_-weighted MRI images were collected for normalization and coregistration with the EPI scan (3D magnetization-prepared rapid gradient-echo sequence, TR = 2,150 ms, TE = 3.5 ms, TI = 1,100 ms, flip angle = 8°, FOV = 256 × 256 × 160 mm^3^, 160 axial slices, resolution = 1 mm^3^).

### Associative Learning and Memory Paradigm

The specifically curated associative learning task cycled through four successive conditions: Memory Formation (Encoding), Post-Encoding Consolidation, Memory Retrieval, and Post-Retrieval Consolidation (27-s conditions each). During Memory Formation, nine objects each uniquely associated with a location within a 3 × 3 spatial grid were presented for naming and encoding (3 s/pair). A stimulus-free (and instruction-free) Post-Encoding Consolidation condition followed (Ravishankar et al., 2019). Following Post-Encoding Consolidation, memory for the nine object-location pairs was tested in a Memory Retrieval condition during which each of the nine grid locations were cued in random order. Participants were required to name the object associated with the location (or utter “no” if they could not recall the object). Finally, a Post-Retrieval Consolidation period followed (which was identical in its form to the Post-Encoding Consolidation period). The task was conducted without feedback, and eight epochs of this sequence of conditions were employed to promote asymptotic performance (a schematic of the task along with observed behavioral data is provided in Supporting Information Figure S1).

### fMRI Data Processing and Time Series

fMRI data were processed in SPM 12 using standard temporal (slice-time correction) and spatial preprocessing methods. For spatial preprocessing, EPI images were oriented to the AC-PC line, corrected for head movement through realignment to a reference image in the sequence, and coregistered to the anatomical high-resolution T_1_ image. Analyses of the displacement parameters indicated that estimated head movement did not differ between groups (.14 ≤ *p* ≤ .98). The deformations from normalizing the high-resolution T1 image were applied to the coregistered EPI images to thus normalize the volumes to stereotactic space. A low-pass filter (128 s) corrected for low-frequency components. At the first level, epochs were modeled with boxcar stimulus functions convolved with a canonical hemodynamic response function to form regressors of interest. In each first level model, the six motion parameters (3 for translation and 3 for rotation) from the coregistration were modeled as covariates of no interest. Images were resliced (2 mm^3^) and a Gaussian filter (8 mm FWHM) applied. Images exceeding 4 mm of movement (<1% of all images) were excised from analyses. The Automated Anatomical Labeling (AAL) atlas was used to identify 90 cerebral regions (nodes) in the a priori network (Rolls et al., 2020).

### Undirected Functional Connectivity

Mean time series were extracted from the 90 cerebral regions for each participant and for each of the four conditions of the task. The bivariate correlations across the full matrix were computed from the time series data in each condition. For each participant, resultant symmetric 90 × 90 adjacency matrices of correlation coefficients (one for each condition of the task) were obtained. These coefficients (Pearson’s *r*) were normalized with the Fisher *Z*-transformation (Equation 1) to ensure the variance is independent of the magnitude of the correlation coefficients (Thompson & Fransson, 2016). The *Z*-values were used for the calculation of graph theoretic metrics.

$$z=\frac{1}{2}\ln\left(\frac{1+r}{1-r}\right)$$ (1)

Supporting Information Figures S2–S5 provide the distance connectomes for each of the four conditions (separately for each of the HC and SCZ groups).

### Betweenness Centrality Analyses and Computation

BC estimates the number of shortest functional paths that traverse through a node. The length between nodes is reflected in the weighted measure of the Fisher *Z*-transformation, which calculates the functional proximity between nodes. Through these estimates, BC represents a node’s role in transmitting and facilitating interactions (van den Heuvel et al., 2010). The BC of a node in the network was computed based on the following formula (Equation 2).

$${BC}^{\text{weighted}}=\frac{1}{\left(n-1\right)\left(n-2\right)}\Sigma_{h\neq i}\frac{{sp}_{hj}\left(i\right)}{{sp}_{hj}}$$ (2)

The expression *sp*_*hj*_(*i*) indicates the number of shortest paths between nodes *h* and *j* that passes through node *i*. BC was estimated in each participant across the 90 cerebral nodes and for each condition. The resultant BC values were forwarded for statistical analyses. Two-sample *t* tests were conducted on the BC values for each node in each condition to study inter-group differences. All patient-control comparisons were thresholded (*p*_FDR_ < 0.05) (Benjamini & Hochberg, 1995) across the 360 conducted tests, to identify nodes with significant inter-group differences.

## ACKNOWLEDGMENTS

We acknowledge the help of all the participants (patients and controls) and of participant families in the conduct of this research.

## SUPPORTING INFORMATION

Supporting Information for this article is available at https://doi.org/10.1162/netn_a_00278.

## AUTHOR CONTRIBUTIONS

Emmanuel D. Meram: Conceptualization; Formal analysis; Investigation; Visualization; Writing – original draft; Writing – review & editing. Shahira Baajour: Conceptualization; Methodology; Resources; Software. Asadur Chowdury: Conceptualization; Formal analysis; Methodology; Resources; Software. John Kopchick: Methodology; Software; Writing – review & editing. Patricia Thomas: Investigation; Project administration. Usha Rajan: Investigation; Project administration; Validation. Dalal Khatib: Data curation. Caroline Zajac-Benitez: Project administration. Luay Haddad: Data curation. Alireza Amirsadri: Data curation; Resources. Jeffrey A. Stanley: Funding acquisition; Investigation; Methodology; Software. Vaibhav A. Diwadkar: Conceptualization; Formal analysis; Funding acquisition; Investigation; Methodology; Project administration; Resources; Software; Supervision; Writing – original draft; Writing – review & editing.

## FUNDING INFORMATION

Vaibhav A. Diwadkar, National Institute of Mental Health (https://dx.doi.org/10.13039/100000025), Award ID: MH111177. Vaibhav A. Diwadkar, Ethel and James Flinn Foundation (https://dx.doi.org/10.13039/100005161). Vaibhav A. Diwadkar, DMC Foundation (https://dx.doi.org/10.13039/100017538). Vaibhav A. Diwadkar, Cohen Neuroscience Endowment. Vaibhav A. Diwadkar, Jack Dorsey Endowment. Vaibhav A. Diwadkar, Lycaki-Young Funds from the State of Michigan.

## TECHNICAL TERMS

Network repertoire: A property of a network as (in our case) summarized by graph theoretic measures.
Learning: The process of associating pairs of arbitrary memoranda during an experimentally titrated paradigm.
Betweenness centrality: Our employed graph theoretic metric indexing a node’s integrative value or “hubness” (i.e., relative importance within a network).
Memory Formation/encoding: A learning condition during which pairs of items are visually presented for forming associations.
Post-Encoding Consolidation: An instruction-free covert condition that immediately follows Memory Formation.
Memory Retrieval: A condition during which participants must recall (from memory) the item associated with the retrieval cue.
Post-Retrieval Consolidation: An instruction-free covert condition that immediately follows Memory Retrieval.
Stability: How much a node’s rank by betweenness centrality changes across conditions (derived from the standard deviation of ranks).
Rank: The order of nodes based on its betweenness centrality; a lower number reflects higher rank (i.e., greater integrative importance).
