# Supplementary material for: The topology, stability, and instability of learning-induced brain network repertoires in schizophrenia
Source: Netw Neurosci. 2023 Jan 1;7(1):184–212. doi: 10.1162/netn_a_00278 (PMC10270714; doi:10.1162/netn_a_00278)
Supplement: Supplementary file 1 [file netn-7-1-184-s001.pdf]

Meram, E. D., Baajour, S., Chowdury, A., Kopchick, J., Thomas, P., Rajan, U., Khatib, D., Zajac-Benitez, C., Haddad, L., Amirsadri, A., Stanley, J. A., & Diwadkar, V. A. (2023). The topology, stability, and instability of learning-induced brain network repertoires in schizophrenia. *Network Neuroscience,* 7(1), 184–212. https://doi.org/10.1162/netn_a_00278

Supplementary Figure 1

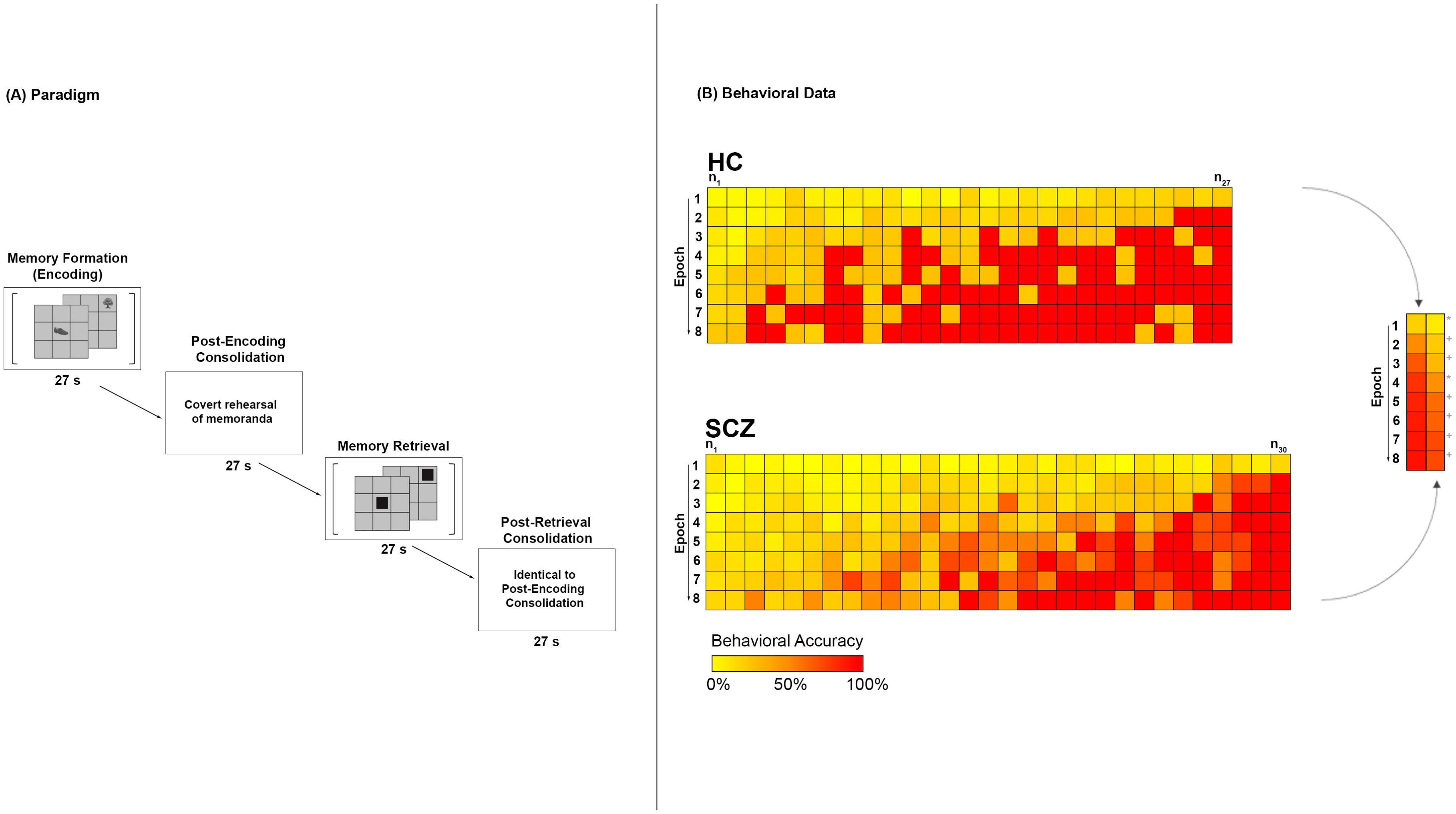

Supplementary Figure 2

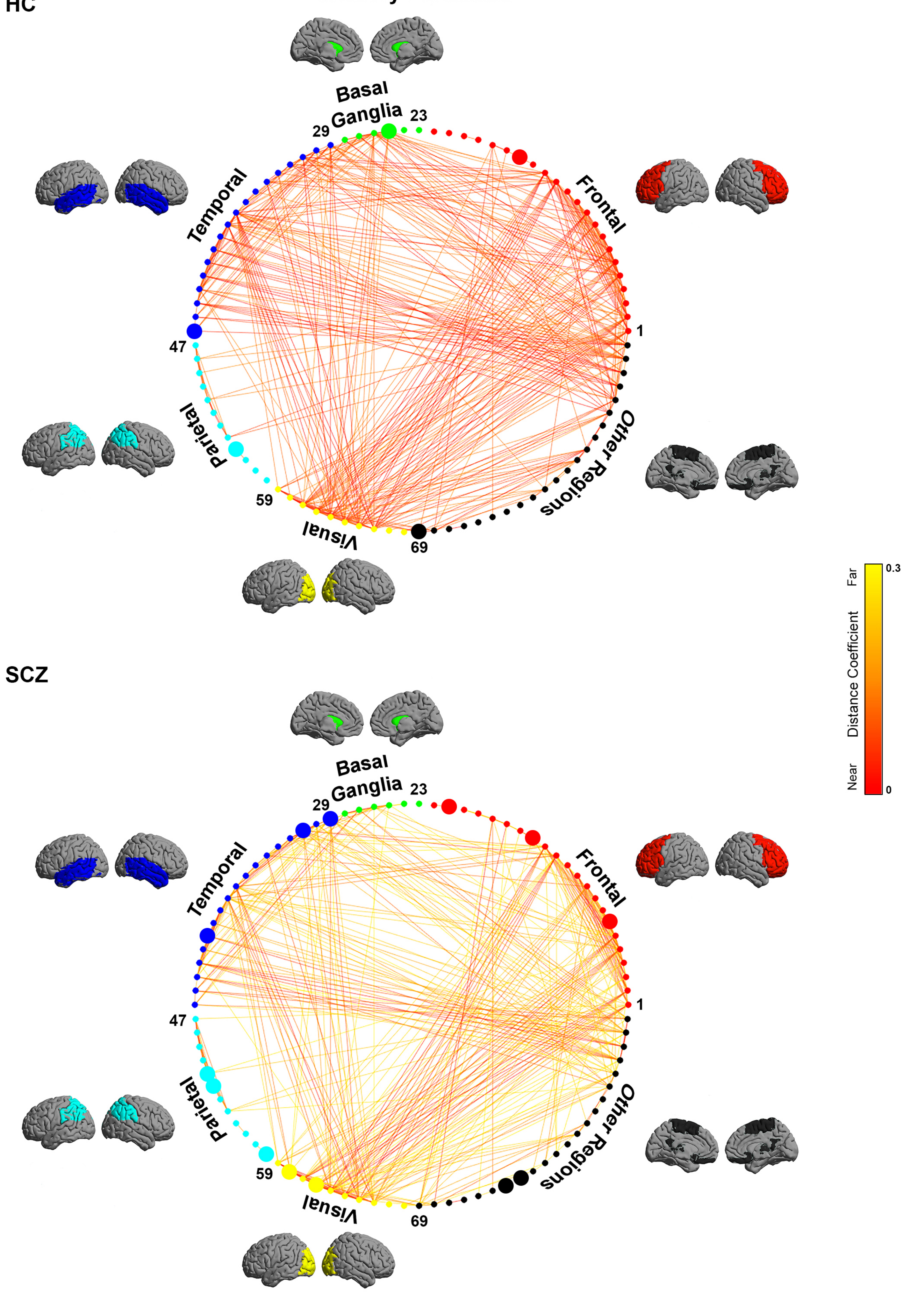

Supplementary Figure 3

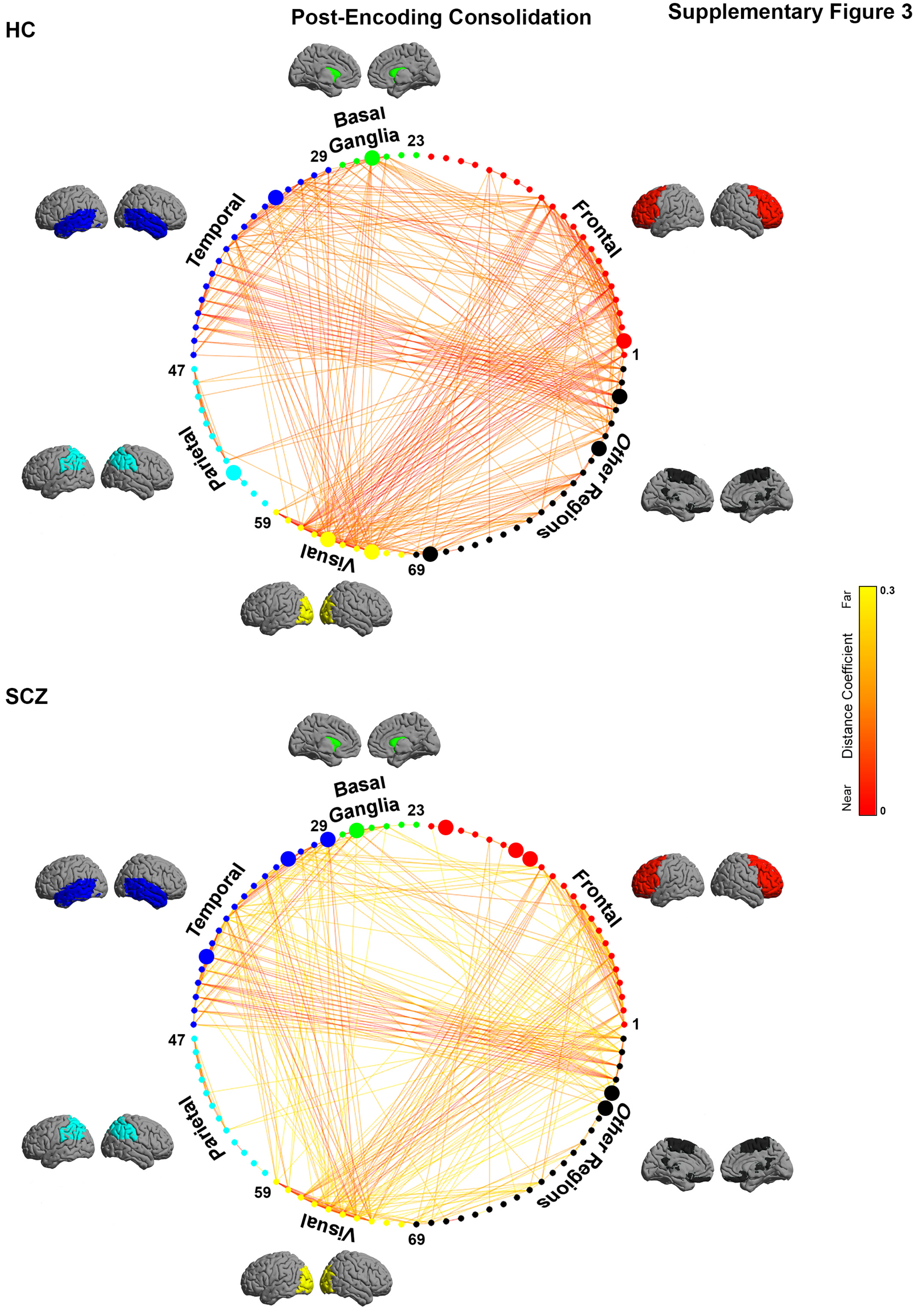

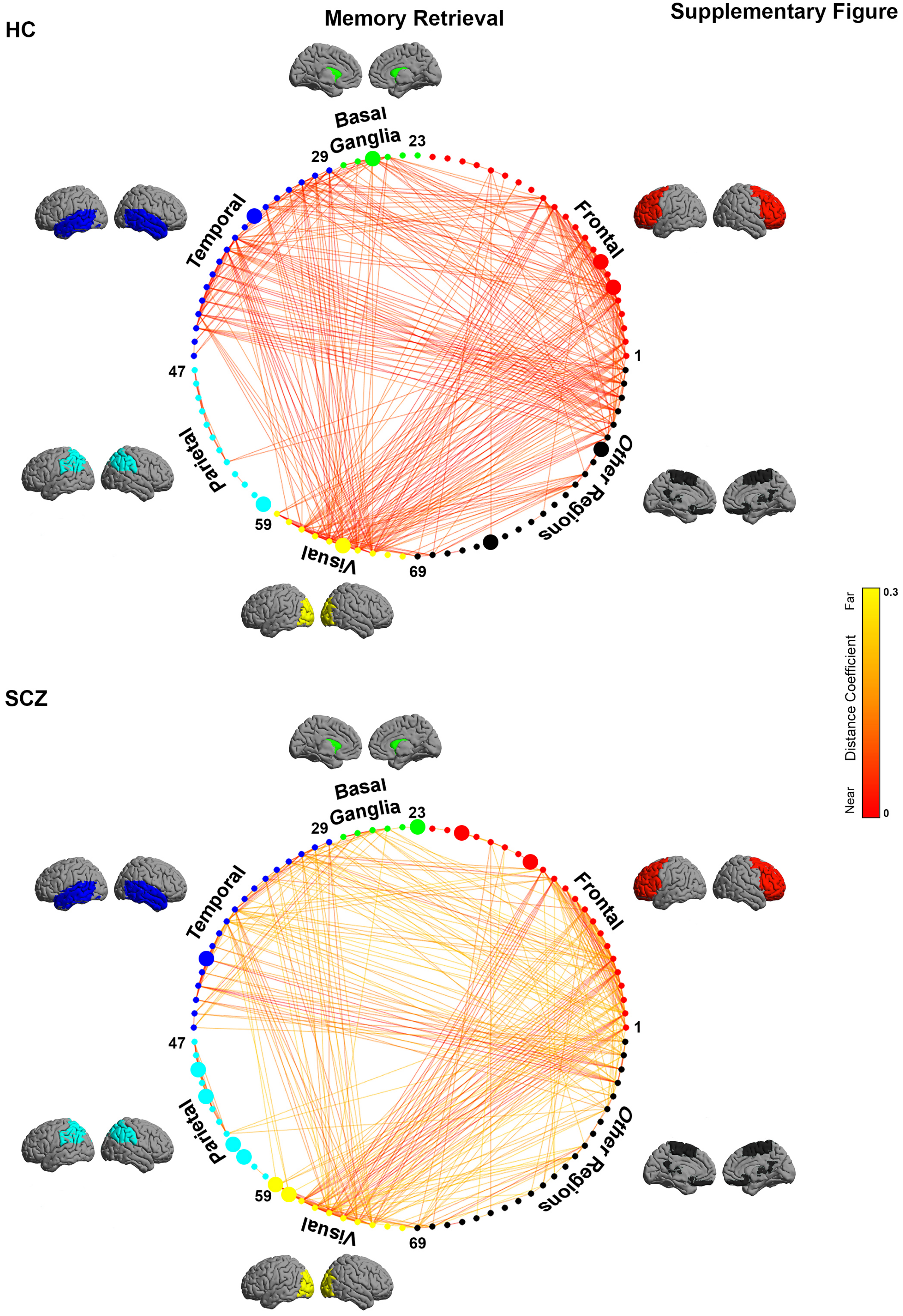

HC
Memory Retrieval
Basal Ganglia
23
29
Frontal
Temporal
1
47
Parietal
Other Regions
59
Visual
69
SCZ
Basal Ganglia
23
29
Frontal
Temporal
1
47
Parietal
Other Regions
59
Visual
69
Far
0.3
Distance Coefficient
Near
0

Supplementary Figure 5

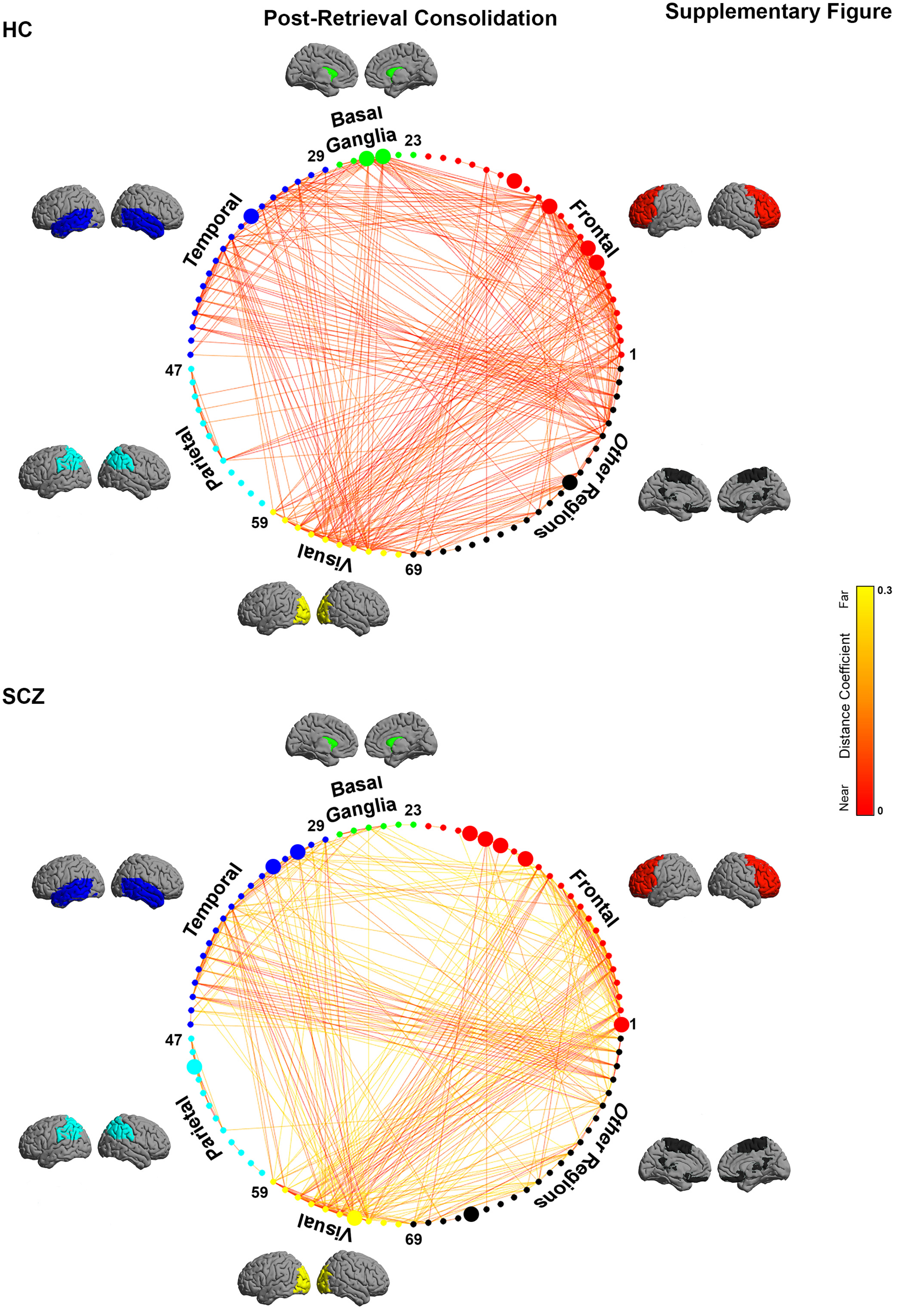

Supplementary Figure 6

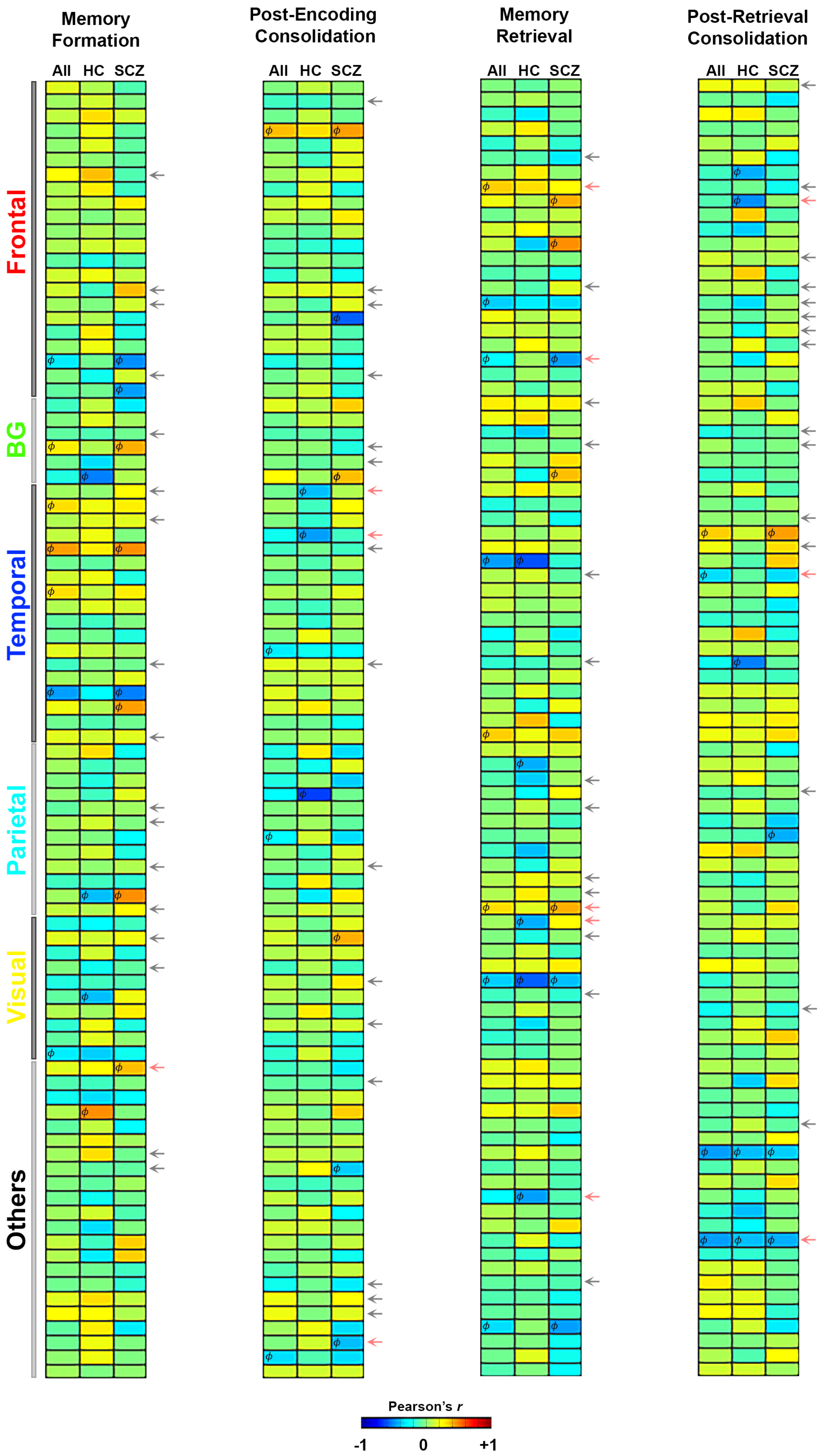

Supplementary Figure 7

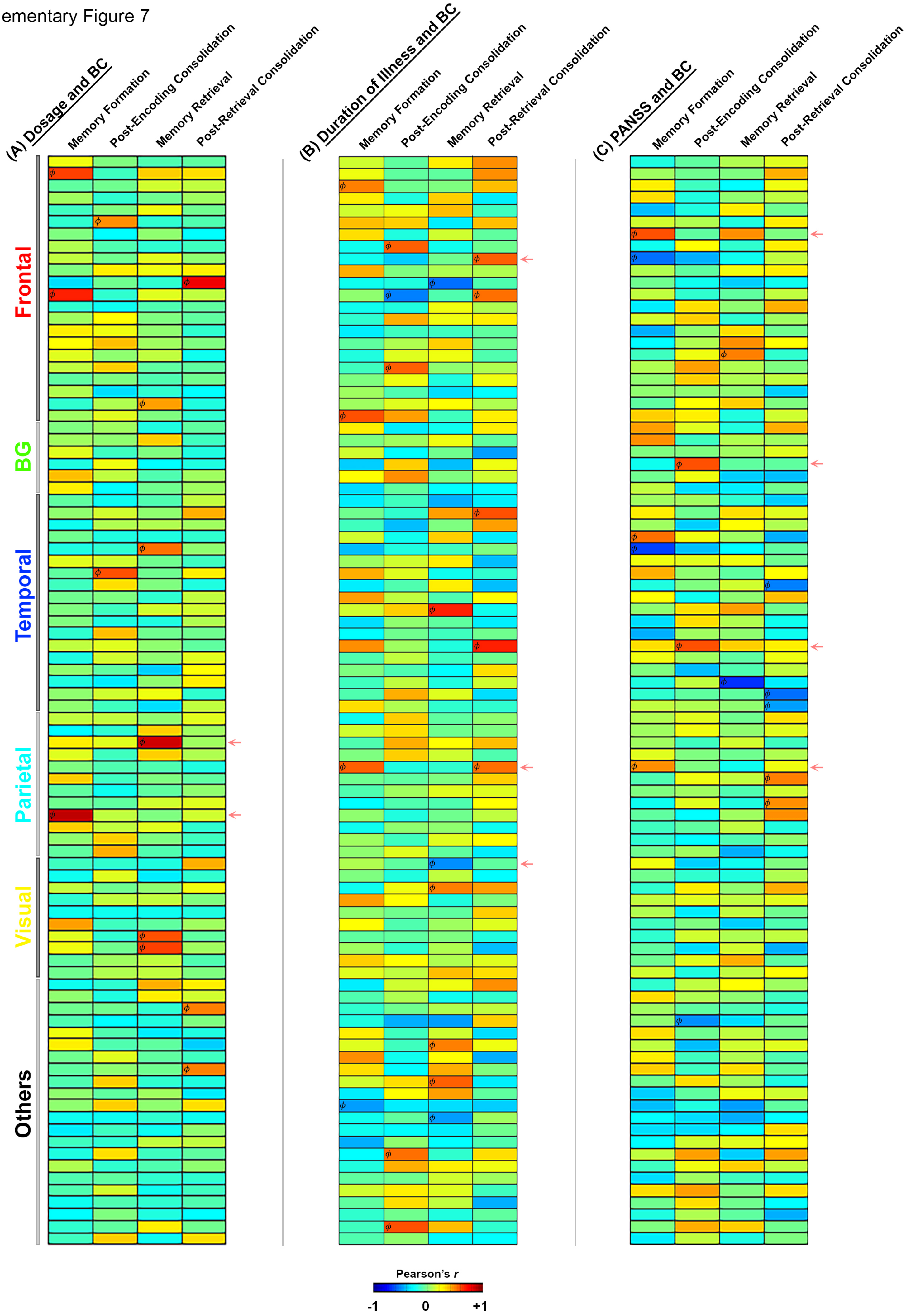

Supplementary Table 1

90 Cerebral Nodes

1. (L) Superior Frontal Gyrus
2. (R) Superior Frontal Gyrus
3. (R) Frontal Superior Medial Gyrus
4. (L) Frontal Superior Medial Gyrus
5. (L) Middle Frontal Gyrus
6. (R) Middle Frontal Gyrus
7. (L) Inferior Frontal Triangularis
8. (R) Inferior Frontal Triangularis
9. (L) Inferior Frontal Operculum
10. (R) Inferior Frontal Operculum
11. (L) Anterior Cingulate Cortex
12. (R) Anterior Cingulate Cortex
13. (L) Middle Cingulate Cortex
14. (R) Middle Cingulate Cortex
15. (L) Superior Frontal Gyrus (Orbital)
16. (R) Superior Frontal Gyrus (Orbital)
17. (L) Medial Frontal Gyrus (Orbital)
18. (R) Medial Frontal Gyrus (Orbital)
19. (L) Middle Frontal Gyrus (Orbital)
20. (R) Middle Frontal Gyrus (Orbital)
21. (R) Inferior Frontal Gyrus (Orbital)
22. (L) Inferior Frontal Gyrus (Orbital)
23. (L) Caudate Nucleus
24. (R) Caudate Nucleus
25. (L) Putamen
26. (R) Putamen
27. (L) Pallidum
28. (R) Pallidum
29. (L) Hippocampus
30. (R) Hippocampus
31. (L) Parahippocampal Gyrus
32. (R) Parahippocampal Gyrus
33. (L) Inferior Temporal Gyrus
34. (R) Inferior Temporal Gyrus
35. (L) Fusiform Gyrus
36. (R) Fusiform Gyrus
37. (L) Amygdala
38. (R) Amygdala
39. (L) Superior Temporal Pole
40. (R) Superior Temporal Pole
41. (L) Superior Temporal Gyrus
42. (R) Superior Temporal Gyrus
43. (L) Middle Temporal Pole
44. (R) Middle Temporal Pole
45. (L) Middle Temporal Gyrus
46. (R) Middle Temporal Gyrus
47. (L) Superior Parietal Lobule
48. (R) Superior Parietal Lobule
49. (L) Inferior Parietal Lobule
50. (R) Inferior Parietal Lobule
51. (L) Supramarginal Gyrus
52. (R) Supramarginal Gyrus
53. (L) Angular Gyrus
54. (R) Angular Gyrus
55. (L) Precuneus
56. (R) Precuneus
57. (L) Cuneus
58. (R) Cuneus
59. (L) Superior Occipital Gyrus
60. (R) Superior Occipital Gyrus
61. (L) Middle Occipital Gyrus
62. (R) Middle Occipital Gyrus
63. (L) Inferior Occipital Gyrus
64. (R) Inferior Occipital Gyrus
65. (L) Calcarine Sulcus
66. (R) Calcarine Sulcus
67. (L) Lingual Gyrus
68. R) Lingual Gyrus
69. (L) Thalamus
70. (R) Thalamus
71. (L) Gyrus Rectus
72. (R) Gyrus Rectus
73. (L) Olfactory
74. (R) Olfactory
75. (L) Paracentral Lobule
76. (R) Paracentral Lobule
77. (L) Supplementary Motor Area
78. (R) Supplementary Motor Area
79. (L) Precentral Gyrus
80. (R) Precentral Gyrus
81. (L) Postcentral Gyrus
82. (R) Postcentral Gyrus
83. (L) Heschl Gyrus
84. (R) Heschl Gyrus
85. (L) Insular Cortex
86. (R) Insular Cortex
87. (L) Posterior Cinglate Cortex
88. (R) Posterior Cingulate Cortex
89. (L) Rolandic Operculum
90. (R) Rolandic Operculum
